# Clay based heterogeneous catalysts for carbon–nitrogen bond formation: a review

**DOI:** 10.1039/d3ra06358e

**Published:** 2024-02-05

**Authors:** P. Vinoth Kumar, G. Madhumitha

**Affiliations:** a Chemistry of Heterocycles & Natural Product Research Laboratory, Department of Chemistry, School of Advanced Sciences, Vellore Institute of Technology Vellore Tamilnadu India madhumitha.g@vit.ac.in dr.g.madhumitha@gmail.com

## Abstract

Clay and modified clay-based catalysts are widely used in organic transformation. Owing to the interlayer ions and good ion exchange capacity of clay, replacement with another ion and incorporation of different nanomaterials can be done. Due to these significant properties of clay, it can be utilized in the synthesis of various organic compounds. Carbon–nitrogen bonded compounds possess diverse applications in different fields. These compounds are prepared using different solid acid heterogeneous catalysts. This review presents a detailed discussion on clay used for the carbon–nitrogen bond formation reaction, such as the Biginelli reaction and A^3^ and KA^2^ coupling reactions. Additionally, other C–N bond formation reactions using various clay-based catalysts such as bentonite, montmorillonite, hydrotalcite and halloysite clay with various metals, metal oxides, Kegging type heteropoly acid and various nanomaterial incorporated clay heterogeneous catalysts are discussed.

## Abstract 2

In this review, we discussed about clay based heterogeneous catalysts used for carbon–nitrogen bond formation.

## Graphical abstract

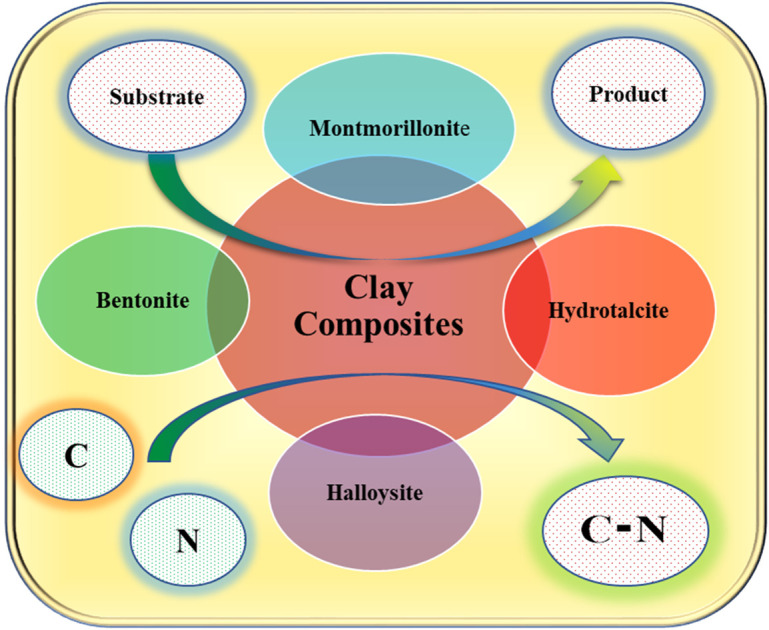

## 1 Introduction

Carbon-heteroatom functionalized compounds have been developed in the past few years; particularly, compounds with C–N bonds are dominant in synthetic chemistry due to their excellent diverse activity in various fields.^1^ Molecules with carbon–nitrogen bonds are abundantly present, such as amino acids, purine and pyrimidine bases and plant extracted alkaloids.^2^ These carbon–nitrogen bonded compounds are most sufficiently present in biologically^3,4^ and pharmacologically active molecules, and they are also present in some natural products, vitamins, drugs, agrochemicals, such as pesticides, herbicides, fungicides,^5^ synthetic intermediates and polymeric materials.^6^ Numerous nitrogen containing heterocyclic moieties are present, and they have excellent applications in various fields; some of these compounds play a predominant role in medicinal chemistry and are used against various health problems as they exhibit medicinal properties, such as anti-cancer,^7,8^ anti-HIV,^9^ anti-bacterial,^10^ anti-viral,^11^ anti-inflammatory,^12^ anti-diabetic,^13^ antiulcer, antioxidant,^14^ and anticonvulsive.^15^ More reactions are available for the synthesis of various C–N formation analogues, such as Cham-Lam coupling,^16–19^ Buchwald coupling,^20–23^ Ullmann coupling,^24,25^ Biginelli reaction,^26,27^ A^3^ coupling,^28^ KA^2^ coupling,^29^ Ugi reaction,^30^ Aza-Michael addition,^31^ amidation,^32^ amino-arylation,^33^ azide-alkene cycloaddition,^34^ Fridlander reaction,^35^ and condensation.^36^ In the past decades, C–N bond formation reactions have been carried out *via* diverse catalysts, such as organic catalysts, metal salts, metal complexes, metal oxides and various homogeneous catalysts. The homogeneous and organic catalysts had high selectivity and provided higher yields. However, they had some limitations pertaining to the recovery of the catalyst and requirement of long reaction time, some tedious ligands and reaction conditions for product formation. To avoid the limitations of homogeneous catalysts, heterogeneous catalysts have been introduced. The use of heterogeneous catalysts has been increasing in recent years over homogeneous catalysts, especially in the field of organic synthesis, and they have excellent activity and specific selectivity for the preparation of desired compounds. However, some heterogeneous catalysts have drawbacks owing to their toxic nature, and the recovery of the catalyst from the reaction mixture requires a very tedious procedure. A minute amount of the catalyst in the product affects the nature of the product, and high energy is required to discard the catalyst. Accordingly, green catalysts play a crucial role in chemical transformation, and the major advantages of green catalysts are less energy consumption, low waste formation, eco-friendly, non-hazardous nature, easily recoverable, and high atom economy.^37–42^ Clay minerals are naturally available inexpensive materials that are heterogeneous in nature, and they do not affect the environment if any chemical reaction is carried out. Various types of clay minerals lead to highly selective products at better yields.^43^ The clay minerals contain hydrated aluminosilicates with nanostructure layers; they possess both Brønsted and Lewis acidic nature due to which they possess excellent activity against various chemical reactions.^44^ Owing to the ultrathin interlayer structure of the clay minerals with alternative cationic and anionic species present on the surface that are easily exchangeable, the clays are classified into two types owing to the ion-exchange property: cationic and anionic clay.^45^ Both positive and negative charges are compensated by the presence of water molecules and ions (Na^+^, Al^3+^, Mg^+^, and Fe^3+^) in the interlayer.^46^ Each aluminosilicate sheet in the clay must contain Si–O tetrahedral and Al–O octahedral sheets in an alternative arrangement like a sandwich. Different types of layered structures are present, for example, the (1 : 1) type possesses one Si–O tetrahedral sheet and one Al–O octahedral sheet alternatively. Additionally, the kaolin type of clay has (1 : 1) and (2 : 1) type; the (2 : 1) layer type has one Al–O octahedral sheet between two Si–O tetrahedral sheets, and the montmorillonite clay has a (2 : 1) type structure in the alternative sandwich model.^47^ The ions present in the clay strongly hold each other by Van der waals, electrostatic and powerful hydrogen bonding forces. The ions present in the clay sheets can be interchanged (cations and anions) (Fe^2+^, Al^3+^, Mg^2+^, Ca^2+^, Na^+^, SO_4_^2−^, Cl^−^, and NO^3−^) without requiring any special process. During the interchanging of ions, the structured nature of nanosheets remains unchanged.^48^ The amount of water molecules present in clay minerals determines the acidic nature of the peculiar clay. In general, clay exhibits Lewis and Brønsted acidity when heated above 300 °C. By increasing the temperature to 450 °C, it becomes anhydrous. Finally, the powder form of clay possesses an excellent Lewis acidic nature because the Brønsted acidity depends on the hydrogen ions (H^+^) present in the compounds.^49^ Clay catalysts are used in various fields, such as semiconductors, dye degradation, supercapacitors, and in the medicinal field.^50,51^ Owing to the clay mineral's high surface area, high ion-exchange capacity, acidity, and easy exchangeability of ions, clay minerals are the cheapest easily available minerals. In recent years, clay-based materials have been developed.

**Fig. 1:**
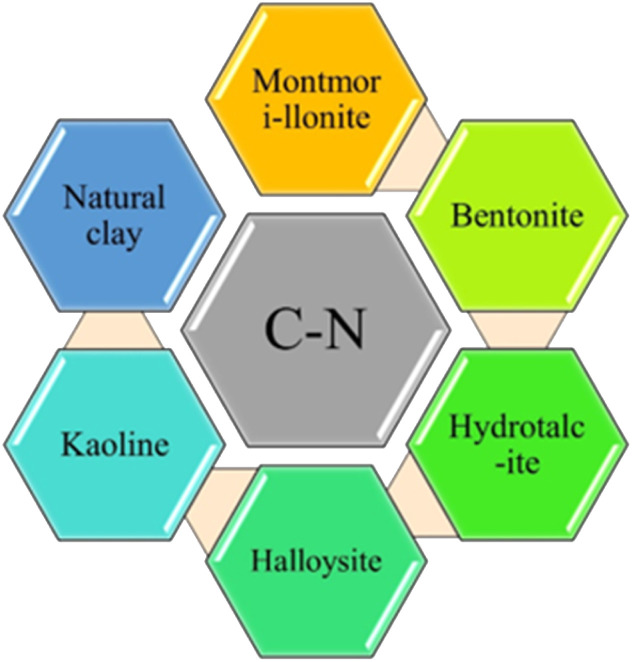
Illustration of the construction of various C–N bond forming compounds using various clay catalysts.

Various clay-based materials have been reported, and the metal and metal oxide incorporated clay material, Kegging type heteropoly acid supported clay substance, and different types of nanoparticle-supported clay materials are nowadays used for diverse applications (Fig. 1). All types of clay must have specific activity and play a crucial role in product formation.

### 1.1 Montmorillonite

**Fig. 2:**
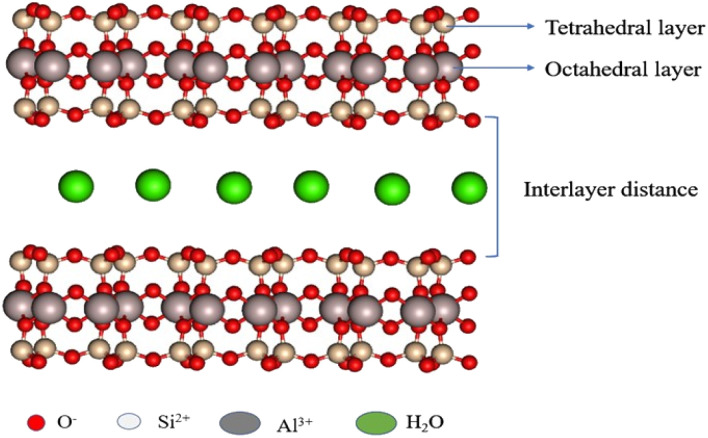
Structure of montmorillonite.

The montmorillonite clay has aluminosilicate (2 : 1) with two tetrahedral sheets (Si–O) in between one octahedral (Al–O) layer (Fig. 2). It comes under the smectite group.^52^ The molecular formula of montmorillonite is Al_2_Si_4_O_10_ (OH)_2_. *n* H_2_O; it has a very good cation exchange property, and its isomorphic substitution occurs between octahedral and tetrahedral-binded cations. The tetrahedral Si^4+^ is replaced by trivalent metal ions, such as Al^3+^; octahedral Al^3+^ is replaced by divalent metal ions, such as Mg^2+^; and when Mg^2+^ ions are introduced in the octahedral layer, the negative charge generated is compensated by Li^+^, Na^+^, and Ca^2+^ ions. This ion-exchange property of montmorillonite clay helps the metal complex, metal nanoparticles, and some organic molecules bound in layers.^53,54^ The clay has both Brønsted acidity and Lewis acidity nature and excellent ion–exchangeable properties.

Owing to the ion-exchangeable property of montmorillonite, sodium and calcium montmorillonites are formed naturally. The physical and chemical properties of clay depend on the interlayer cations. Each layer of the montmorillonite possesses a 1 nm thickness and the breadth of the sheets ranges from 100 to 1000 nm range.^55,56^ The major advantages of montmorillonite are that it is easily available, non-toxic, does not affect the environment, possesses both Lewis and Brønsted acidity nature, has a high surface area, has high pore volume, has high swelling, is expendable in nature, and is easily recoverable and reusable.^57,58^

### 1.2 Bentonite

**Fig. 3:**
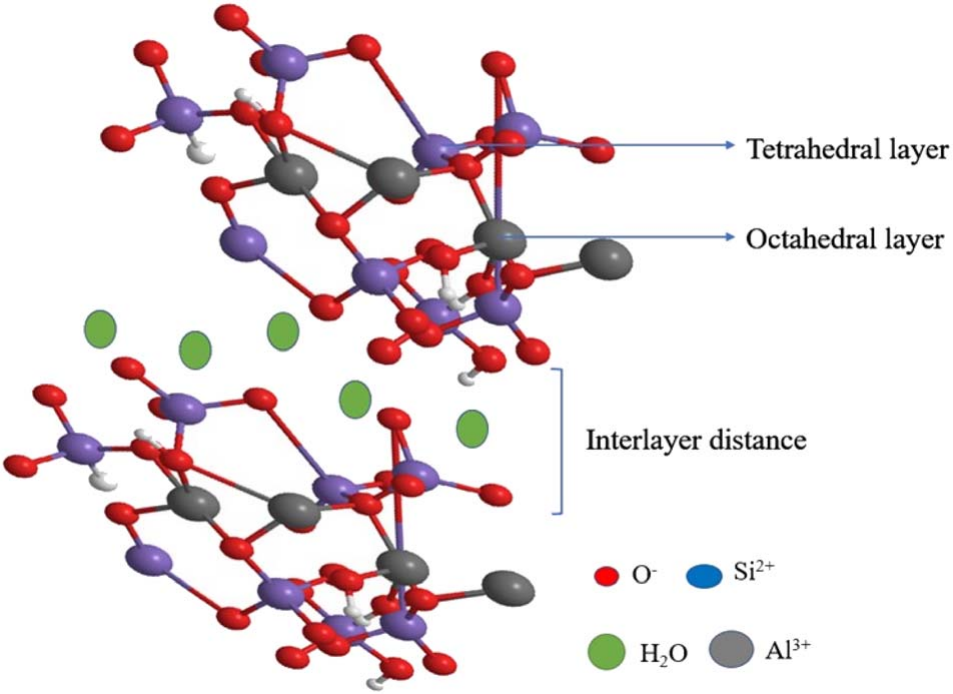
3D structure of bentonite clay.

Bentonite is a natural clay that is classified under the smectite family. The molecular formula of the bentonite is Rx(H_2_O)_4_ {Al_2_ x, Mgx)_2_[(Si, Al)_4_O_10_](OH)_2_}, where a major component of the bentonite contains montmorillonite clay (Fig. 3). It possesses an excellent cation exchange capacity that depends on the interlayer adsorbed cations, such as calcium bentonite and sodium bentonite. Additionally, bentonite has an excellent swelling capacity and low-hydraulic conductivity. The adsorptive capacity of acid-activated bentonite clay possesses excellent catalytic activity owing to its high surface area and porosity. Owing to the hydrophobic nature of clay, it is used in various fields for the purification of water, and bentonite is also a good adsorbent when used as an additive. It is cheap, easily available, has a high surface, and is highly porous, and it can be used as a catalyst in various organic syntheses.^59–62^

### 1.3 Hydrotalcite

**Fig. 4:**
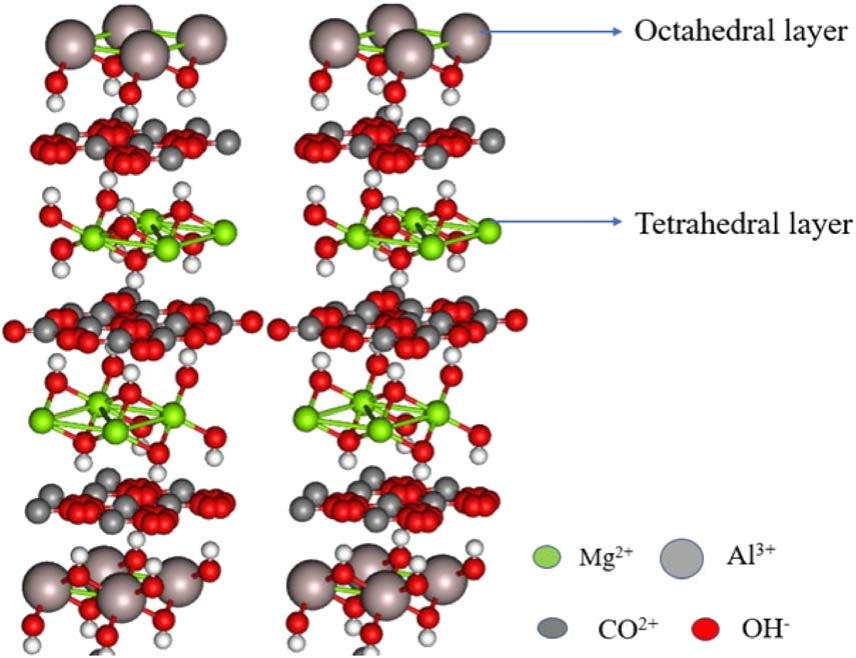
Structure of hydrotalcite clay.

Hydrotalcite is an anionic clay that consists of carboxylate and hydroxyl groups in its interlayer lattice. The molecular formula of hydrotalcite is Mg_6_Al_2_(OH)_16_CO_3_·4H_2_O. The hydrotalcite contains a brucite-type positive layer structure of Al^3+^ or Mg^2+^ ions, and negatively charged anions are bound between the water molecules (Fig. 4). It has excellent ion-exchange properties. The cations of the hydrotalcite can be exchanged using similar ionic radii metal ions, and the anions of the hydrotalcite can be exchanged using different anionic species and metal complexes (Cl^−^, Br^−^, and I^−^).^63–65^ The hydrotalcite clay possesses high adsorption capacity, high thermal stability, high surface area and tunable basicity. Owing to this nature, hydrotalcite clay is used in many fields, especially in medicinal applications. The clay exhibits excellent activity when hydrotalcite is activated and supported by metal nanoparticles or Kegging type heteropoly acids.^66,67^

### 1.4 Halloysite

**Fig. 5:**
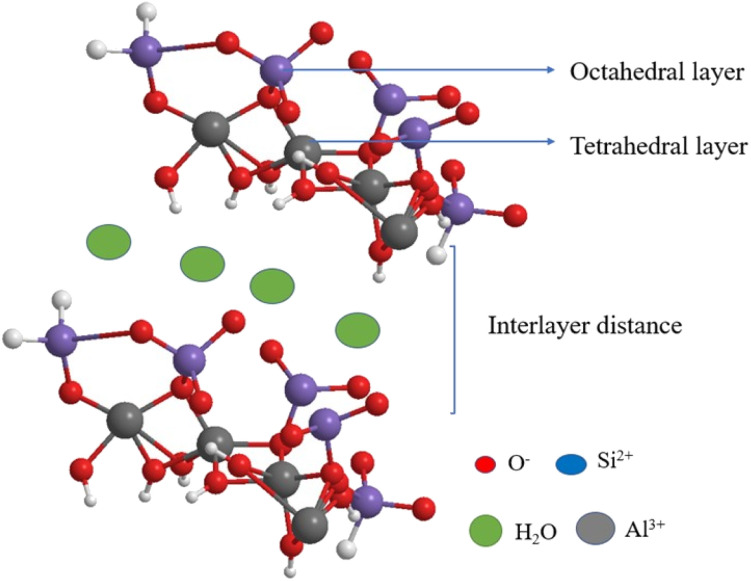
3D structure of halloysite.

Halloysite nano clay is naturally available hydrated aluminosilicates belonging to the kaolinite group of clay. The molecular formula of halloysite clay is Al_2_(OH)_4_Si_2_O_5_·2H_2_O. The structure and reactive nature of Halloysite nanotubes are similar to those of kaolinite clay.^68^ The water molecule present in the halloysite separates each monolayer; the space between each layer is 10 Å. The SiO_4_ moiety is located on the outer surface of the HNT clay, and the Al (OH)_3_ layer is located on the inner surface of the clay.^69^ Tetrahedral SiO_4_ and octahedral Al (OH)_3_ sheets are bound to each other alternatively with water molecules in between separating each layer (Fig. 5). It possesses various morphologies, but the elongated tube type morphology has better activity. It has excellent applications in various fields owing to its high surface area, high thermal and mechanical strength and excellent ion-exchange capacity.^70^ The HNTs have recently been used in drug delivery, photo-degradation, sewage treatment, and electrical and optical applications.^71^ The main merits of the HNTs are their biocompatibility, ease of availability, inexpensiveness, eco-friendliness and easy recyclability, and they do not require any tedious procedure.^72^

### 1.5 Kaolin

**Fig. 6:**
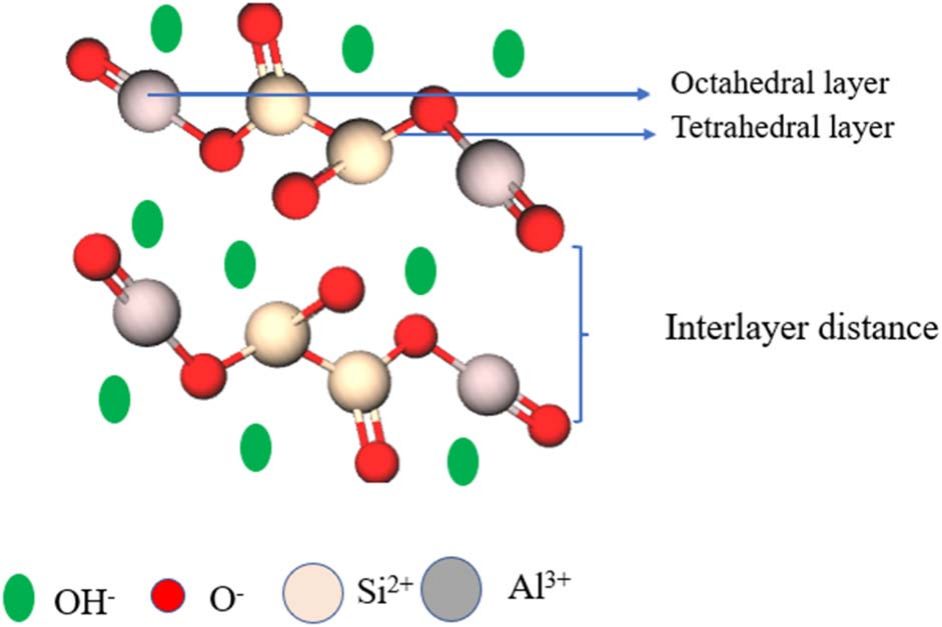
3D structure of kaolin clay.

Kaolin is another naturally and commercially available heterogeneous solid acid; it has a 1 : 1 layer of aluminosilicates, and the tetrahedral SiO_4_ and octahedral AlO_2_ (OH)_4_ groups are arranged in a sandwich.^73^ The Al–O groups are bound between two tetrahedral Si–O moieties and the water molecule is located in the interlayer of each sandwich moiety. The molecular formula of kaolin clay is Al_2_Si_2_O_5_(OH)_4_; the oxygen atom of tetrahedral Si–O is boned with the octahedral aluminum atom, and one oxygen atom binds with only one Al^3+^ moiety (Fig. 6). It has a high surface area, strong acidic nature, and excellent thermal stability property. Owing to its diverse properties, kaolin is used in various fields to provide excellent yield.^74–76^

In 2011, Nagendrappa briefly encapsulated the various clay-based catalysts used for organic synthesis: up to 2010.^77^ In 2012, Kaur summarized the montmorillonite clay-based catalyst used in various types of organic reactions up to 2012.^78^ Next, Kumar and his group documented the montmorillonite K10 and different moieties attached MMT-K10 clay catalyst used in organic reactions up to 2014.^79^ Dutta in 2020 published a metal nano-particle supported montmorillonite clay used in organic transformation, and synthesis of metal nano-particle supported montmorillonite was discussed detailly.^80^ In 2021, Nagendrappa briefly summarized the clay and clay-based materials used for organic syntheses, such as Biginelli reactions, condensation, addition, oxidation and reduction reactions.^81^ In our research group, Chellapandi in 2021 detailly discussed the montmorillonite clay-based catalyst used for the synthesis of various N-heterocycles, such as five and six-member heterocycles.^82^

Organic transformations are mimics of the natural products. It involves the construction of target molecules from small entities. Hetero atom-attached compounds exhibit excellent activity, and C–N bonded compounds are used in medicinal, agricultural and sensor fields. Nowadays, many C–N-coupled organic compounds are available owing to their selective applications in various fields. In Table 1, we discuss the various existing methods of C–N bond formation reactions with their reactivity. Bariwal and his research group in 2013 briefly summarized a C–N bond formation cross-coupling reaction.^83^ Ghorai *et al.* in 2017 summarized an iron-based catalyst used in C–N bond formation reactions.^84^ In 2018, Karkas reported a summarized work of C–N bond formation *via* electrochemical methods.^85^ Xia and the research group in the same year reported a summarized work of C–N bond formation using radical-based photo/electro chemistry methods.^86^ Kaur *et al.* in 2019 briefly summarized the C–N bond formation reaction using a ruthenium-based catalyst for the synthesis of five-membered N-heterocycles.^87^ Bharatam and his research group in 2020 summarized the synthesis of drugs and biorelevant N-heterocycle C–N bond formation.^88^ Schomaker *et al.* in 2021 reported a briefly summarized work of enantioselective C–N bond formation *via* nitrene transfer catalyst.^89^

**Table 1:** Some existing methods of C–N bond formation

S. no	Type of reaction	Catalyst	Product	Yield	Ref.
1	Cham–Lam coupling	Copper iminoarylsulfonate complexes	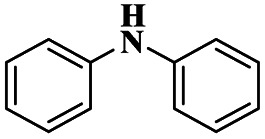	99%	16
		[Cu(DMAP)4I]I complex	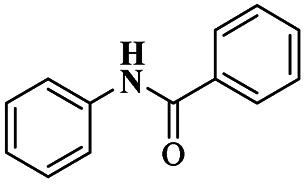	90%	17
		CuI	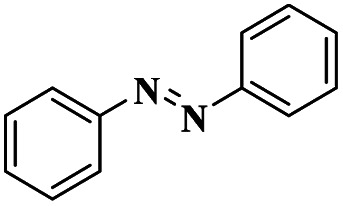	92%	18
		NiCl_2_·6H_2_O	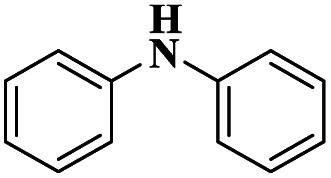	85%	19
2	Buchwald–Hartwig cross-coupling	[Pd(NHC)(allyl)Cl]	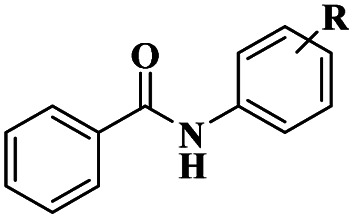	88%	20
		*N*, *N*-symmetrical benzimidazolium clockPd-PEPPSI complex	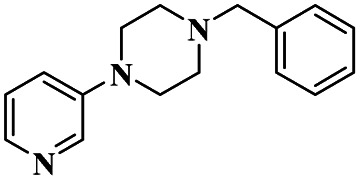	93%	21
		Cp*Co(iii) and Cu(OAc)_2_ bimetallic catalysis	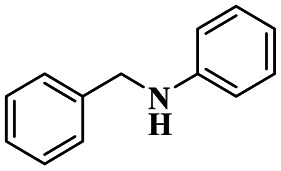	91%	22
		Pd(dba)_2_	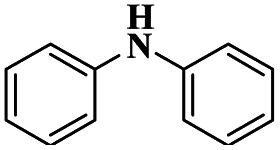	94%	23
3	Ullmann coupling	Cu/Cu_2_O	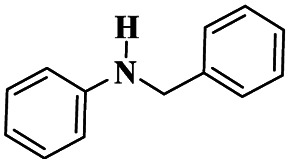	77%	24
		CuI	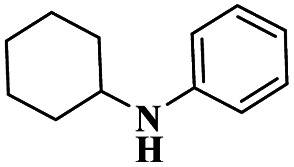	98%	25
4	Biginelli reaction	Yb (4,6-*O*-ethylidene-*N*-(2-hydroxybenzylidene)-β-dglucopyranosylamine) complex	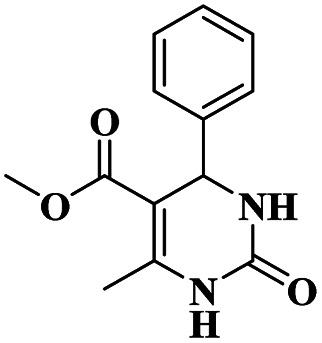	95%	26
		Fe_3_O_4_-bpy-Ni complex	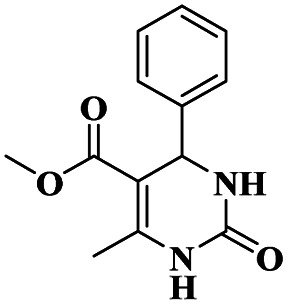	90%	27
5	A^3^ coupling	Ag_2_CO_3_	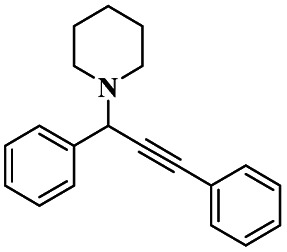	90%	28
6	KA^2^ coupling	Zn(OAc)_2_	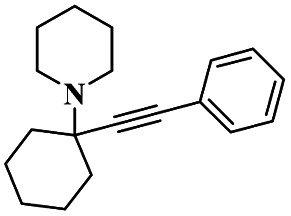	91%	29
7	Ugi reaction	Pd(OAc)_2_	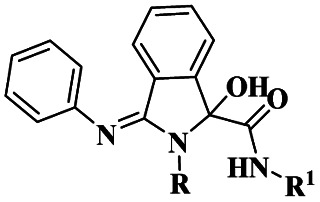	82%	30
8	Aza-Michael addition	[Pd(cinnammyl)Cl]_2_	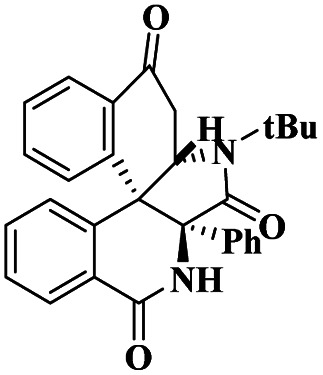	95%	31

In this review, we discuss the clay compound-supported catalyst used for the construction of C–N moieties. Clay and modified materials with synthetic methods and properties are discussed in detail in the following protocols (Table 2).

**Table 2:** Summary of clay-supported carbon–nitrogen bond formation reaction using different modified materials under different synthetic methods

S. no.	Type of clay	Modified material	Solvent	Synthetic approach	Efficiency	Reference
1	Bentonite	Imidazole@Au NP	Ethanol	Conventional heating method	92%	90
2	Bentonite	B-ZVIN	Solvent free	Greener method	96%	91
3	Bentonite	TSA	Ethanol	Reflux	89%	92
4	Bentonite	Fe (iii)	Acetonitrile	Conventional heating method	95%	93
5	Bentonite	Ionic liquid	H_2_O/EtOH	Greener method	98%	94
6	Bentonite	TPA	Ethanol	Conventional heating method	95%	95
7	Halloysite	Amine-F-HPA	Water	Reflux	96%	96
8	Halloysite	HPA-creation	Water	Reflux	95%	97
9	Halloysite	Cu@ furfal imine	Water	Ultrasonication	95%	98
10	Halloysite	Cu-amine-HNT	Ethanol	Ultrasonic method	93%	99
11	Halloysite	Cu-triazole	Water	Ultrasonic method	95%	100
12	Halloysite	HNTs	Solvent free	Reflux	95%	101
13	Hydrotalcite	Mg/Fe	Solvent free	Conventional heating	90%	102
14	Hydrotalcite	—	Water	Reflux	94%	103
15	Hydrotalcite	Cu/Fe	TBMP	Conventional heating	92%	104
16	MK-10	—	HCl/Toluene	Greener method	94%	105
17	MK-10	—	Solvent free	Greener method	85%	106
18	MK-10	—	Solvent free	Greener method	90%	107
19	MK-10	NH_2_	EtOH	Reflux method	98%	108
20	MK-10	—	EtOH	Conventional heating method	82%	109
21	MK-10	—	EtOH	Reflux method	83%	110
22	MK-10	—	Water	Greener method	95%	111
23	MK-10	Metal Schiff base	CH_3_CN	R.T	81%	112
24	MMT	Cu/amine	Water/EtOH	Greener method	95%	113
25	MK-10	Cu_2_O/CuO	Water	Green method	98%	114
26	MK-10	Cu_2_O	Water	Green method	98%	115
27	MMT – KSF	—	Solvent free	Greener method	87%	116
28	MMT – KSF	GC	Solvent free	Greener method	96%	117
29	MMT – KSF	HPA	Solvent free	Greener method	96%	118
30	MMT – KSF	Cu doped	Solvent free	Microwave	98%	119
31	HPVAC-MK10	HPVAC	Solvent free	Greener method	95%	120
32	HPA-MK10	HPA	Solvent free	Greener method	95%	121
33	MMT	CTA-PMo	Solvent free	Greener method	96%	122
34	MMT	VMWP	Solvent free	Greener method	92%	123
35	MMT	PVMoK	Solvent free	Greener method	97%	124
36	MMT-K10	PVMoK-10	Solvent free	Greener method	94%	125
37	Na^+^-MMT	Cu@imine	Solvent free	Greener method	96%	126
38	Na^+^-MMT	[Pmim] HSO_4_	Solvent free	Greener method	90%	127
39	Na^+^-MMT	Perchloric acid	Solvent free	Greener method	91%	128
40	Na^+^- MMT	[Pmim] HSO_4_	Solvent free	Greener method	94%	129
41	MMT	Ag-NP	Toluene	Reflux	95%	130
42	MMT	Acid activated	Ethanol	Reflux	98%	131
43	Nano clay	Zwitter ionic sulfamic acid	Solvent free	Greener method	95%	132
44	Natural clay	HPA	Solvent free	Greener method	93%	134
45	Natural clay	HPVAC-20	Solvent free	Greener method	92%	135
46	Red brick clay	—	Solvent free	Greener method	96%	136
47	KF-clay	—	MeCN	Reflux method	97%	137
48	Kaoline	PMoW	Solvent free	Greener method	95%	138
49	Red clay	—	Solvent free	Greener method	80%	139
50	White clay	—	Solvent free	Greener method	95%	140

### 1.6 Scope of the review

The list of literature discussed in this review was published from 2016 to date. From these studies, the synthesis of compounds with carbon–nitrogen bond in the presence of clay-based catalysts was compiled according to the class of clay along with the published year.

## 2 Discussion

### 2.1 Bentonite clay-based catalyst using carbon–nitrogen bond formation reactions

Gholinejad and his group^90^ addressed the synthesis of propargylamine *via* A^3^ coupling in an aqueous solution. The authors introduced gold nanoparticles to assist imidazole-modified bentonite clay as an efficient catalyst for conducting an A^3^ coupling reaction to prepare propargylamine. Initially, various homogeneous catalysts, including transition metals, were used as catalysts to proceed A^3^ coupling reaction, but those methods had some drawbacks, such as high cost and difficulty recycling the catalyst. The authors used a natural bentonite clay-modified gold nano-particle catalyst. The gold nanoparticle-assisted imidazole-modified bentonite acts as a better catalyst compared with pure bentonite.

**Scheme 1:**
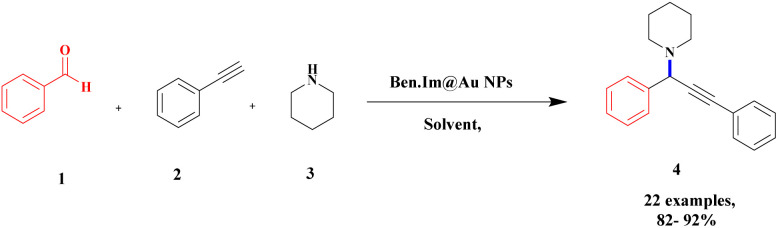
Gold nanoparticle-supported imidazole-modified bentonite clay catalyzed A^3^ coupling.

The reaction between aromatic aldehyde 1, piperidine 2 and phenylacetylene 3 in the presence of Au-supported imidazole-modified bentonite clay catalyst yielded propargylamine 4 product with up to 90% yield (Scheme 1). The same reaction performed in a different catalyst medium provided a very lower yield compared to the bentonite clay-modified catalyst. Additionally, the catalyst was easily separated from the reaction mixture and reused for four consecutive cycles, which provided a better yield.

**Scheme 2:**
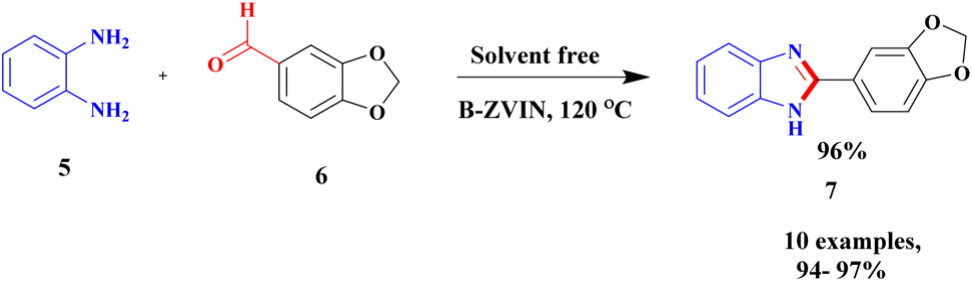
Synthesis of benzimidazole using B-ZVINP.

Sravanthi and group^91^ proposed a facile protocol for the synthesis of benzimidazole *via* bentonite clay-supported zero-valent iron nanoparticles. The authors synthesized a catalyst, B-ZVIN, using a greener method without any hazardous chemicals. An environmentally friendly Eucalyptus leaf extract was used, and during the synthesis process, no toxic by-product was obtained. The zero-valent iron nanoparticles bound with bentonite clay exhibited excellent catalytic activity and provided a better-coupled product. The reaction did not proceed further without bentonite, and the recoverability of the catalyst was tedious. The reaction between *o*-phenylenediamine 5 and aromatic aldehyde 6 under solvent-free conditions was carried out in the presence of a bentonite-supported immobilized zerovalent iron nanoparticle catalyst. The expected product fused to benzimidazole 7 was formed at a better yield of up to 95% (Scheme 2).

**Scheme 3:**
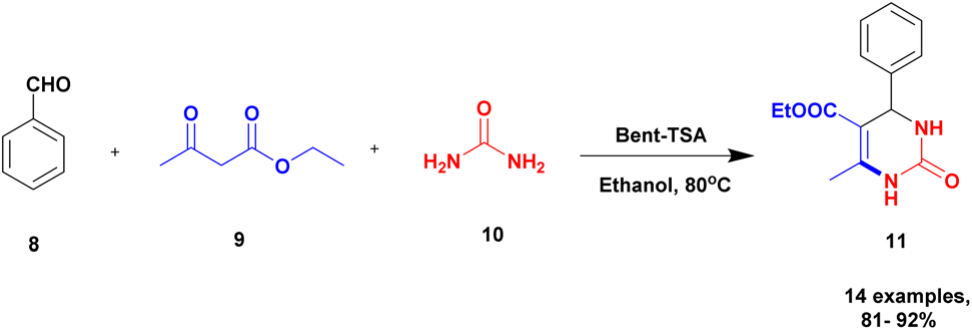
3, 4-Dihydropyrimidine synthesis using 30% TSA-supported bentonite clay.

Chopda and his group^92^ addressed the synthesis of 3, 4-dihydropyrimidine *via* 12-tungstosilicic acid-supported natural bentonite clay used as a catalyst. The reaction was carried out using a one-pot method under acidic conditions. This reaction had few drawbacks, such as the reaction conditions and the difficulty of reclaiming the catalyst. To avoid these limitations, a solid acid-supported heterogeneous catalyst was utilized. The solid-acid supported catalysts are very costly, so the authors introduce easily available bentonite clay-supported solid-acid catalysts. Tungstosilicic acid-supported bentonite has better catalytic activity compared with pure bentonite. Here, the reaction between benzaldehyde 8, ethyl acetoacetate 9, and diamino-ketone 10 in the presence of a small amount of 30%TSA bentonite catalyst formed the dihydropyrimidine 11 product with up to 89% yield. A simple separation technique and normal filtration were used to easily recover the catalyst and reuse it for another set of reactions, for six cycles without loss of its activity and provided better yield (Scheme 3).

**Scheme 4:**
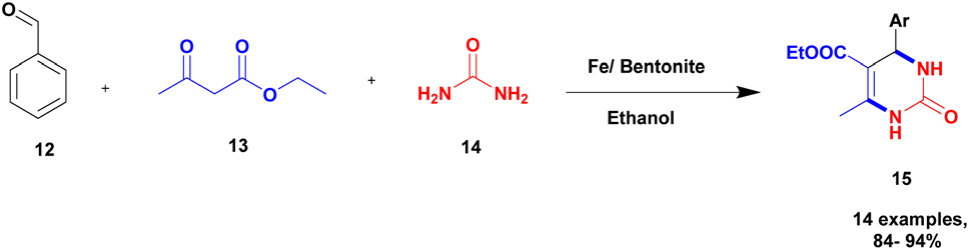
Synthesis of 3, 4-dihydropyrimidine under Fe (iii)/bentonite catalyst.

Chopda and his group^93^ addressed a protocol to carry out the Biginelli reaction *via* Fe(iii)/bentonite clay heterogeneous catalyst. Initially, the Biginelli reaction was performed under different homogeneous catalyst media, and they provided good yields but with some drawbacks. It is very hard to recover the catalyst from the reaction mixture, and some amount of the catalyst remained in the reaction mixture, which affected the formation of the product. To avoid these drawbacks, a heterogeneous catalyst, such as bentonite, was introduced. It is a naturally available clay mineral and possesses a high surface area and an acidic nature. The authors introduced Fe (iii) metal ions incorporated into bentonite clay to increase the acidic nature and improve the catalytic activity. The metal ion-incorporated bentonite composite exhibited excellent catalytic activity. Owing to Fe (iii) binding with clay, *d*-space was increased; Fe (iii) replaced the other metal ions bonded to the bentonite. When compared with pure and incorporated clay, 30% Fe (iii)/bentonite exhibited excellent activity compared with the pure one. The reaction between aromatic aldehyde 12, ethylacatoacetate 13, and diaminoketone 14 in the presence of 30% Fe (iii)/bentonite catalyst produced dihydropyrimidine 15 with a better yield of 90% (Scheme 4). The same reaction without iron gave a poor yield.

**Scheme 5:**
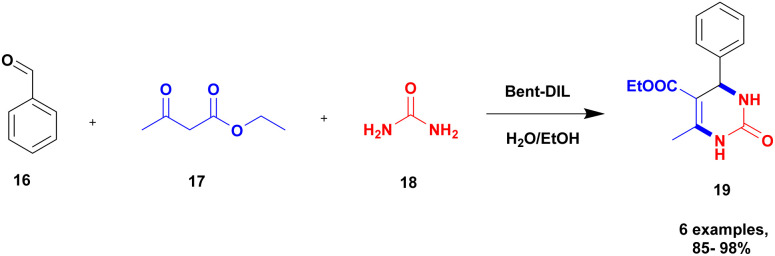
Synthesis of dihydropyrimidinone using a bent-DIL catalyst.

Sadjadi and group^94^ reported a non-metallic ionic liquid-loaded bentonite catalyst for the synthesis of dihydropyrimidinone using a greener method. Initially, the Biginelli reaction was carried out using different catalysts, but metal-free heterogeneous ionic liquid-loaded bentonite was introduced to provide a better yield. When the ionic liquid reacted with the dendritic moiety, a bent-D-IL catalyst was formed, and it has excellent catalytic activity compared to pure bentonite. The dendritic material has more reactive sites to bind with ionic liquid; this leads to the excellent activity of the composite. The reaction between benzaldehyde 16, ethyl acetoacetate 17, and urea 18 in the presence of the bent-D-IL catalyst provided a dihydropyrimidin 19 product with an excellent yield of up to 98% (Scheme 5). The catalyst helps to achieve higher yields. The dendritic moiety effectively improved the ionic loading, leading to an increased catalytic activity.

**Scheme 6:**
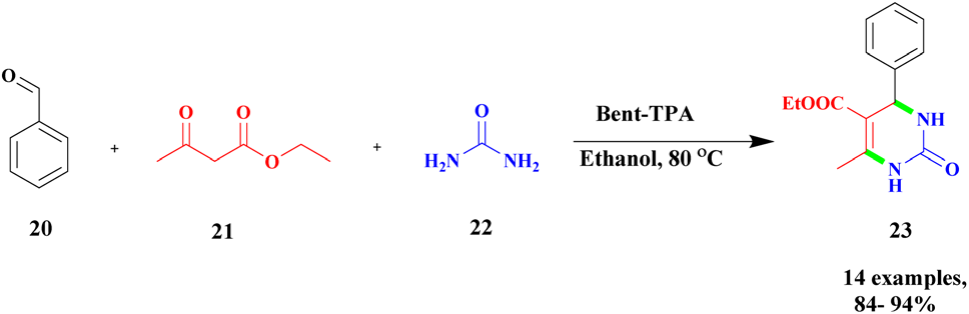
Bent-TPA catalyzed dihydropyrimine synthesis.

The new protocol for the construction of 3, 4-dihydropyrimidine-2-(1*H*)-one *via* heteropoly-12-tungstophosphoric acid-assisted simple bentonite clay composite was used as a heterogeneous catalyst.^95^ Recently, a heterogeneous catalyst was introduced, and it exhibited excellent catalytic activity. Tungsten-based heteropoly acid was used as a heterogeneous catalyst, which provided a better yield. Here, the authors prepared the tungsten-based heteropoly acid-supported bentonite clay catalyst, which exhibited excellent catalytic activity and provided a very good yield compared with pure HTPA up to 92%. The reaction between the aromatic aldehyde 20, ethylacetoacetate 21, and urea 22 in the presence of bent-TPA catalyst and ethanol solvent provided diaminopyrimidinone 23 up to a 95% yield (Scheme 6). The reaction was performed in three different percentages of TPA-loaded bentonite (10%, 20%, and 30%) and carried out with different solvents as well as using a solvent-free method. The solvent-free condition provided a better yield of up to 91%, while the ethanol medium produced a higher yield of up to 95%. The catalyst was reused for five cycles, and it provided better results.

### 2.2 Halloysite clay-based catalyst using carbon–nitrogen bond formation reactions

**Scheme 7:**
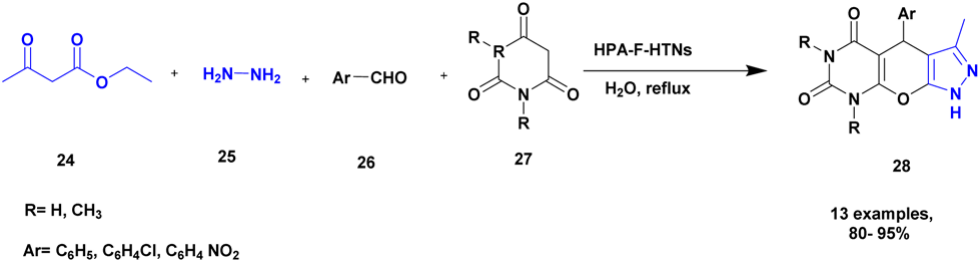
Synthesis of pyrazolopyranopyrimidines by HPA-F-HTN catalyst.

Sadjadi and his group^96^ reported the synthesis of pyrazolopyranopyrimidines *via* hetero polyacid-supported amine-functionalized halloysite clay heterogeneous catalysts. The heteropoly acids showed excellent catalysts, with both Brønsted acidity and better redox properties. Owing to this property, it is used as a catalyst in various organic reactions. Halloysite clay nanotubes have been introduced in recent years for various applications, including drug delivery systems. Owing to the large surface area and specific tunability nature of the halloysite, the heteropolyacid-supported halloysite exhibited excellent activity compared with the pristine one. The author performed a four-component domino reaction between ethyl acetoacetic ester 24, hydrazine 25, aromatic aldehyde 26 and barbituric acid 27 in the presence of heretopolyacid over amine-functionalized halloysite nanoparticle. Finally, it provided pyrazolopyranopyrimidines 28 product with a better yield of up to 96% (Scheme 7). When compared with the other earlier methods, the HPA-F-HTNs in water used as solvent provide a better yield. The catalyst in the reaction mixture was easily separated and reused without loss of its activity, even after three cycles with up to 90%. The major advantages of the catalyst were the short reaction time in an aqueous medium and the reusability of the catalyst.

**Scheme 8:**
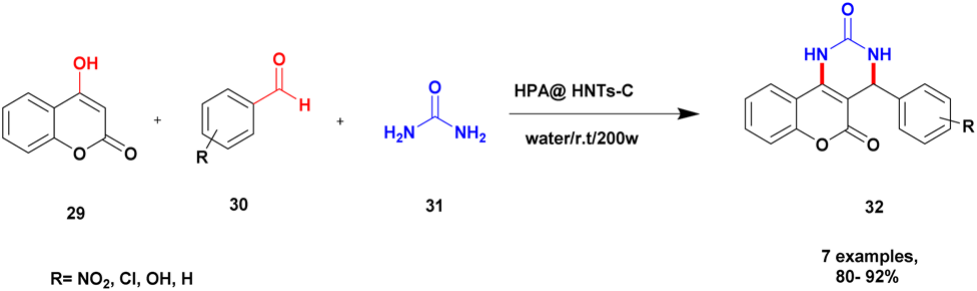
Synthesis of benzopyranopyrimidine *via* HPA@creatinHNT catalyst.

A novel heteropoly acid-incorporated creation functionalized halloysite clay heterogeneous catalyst was prepared and used for the synthesis of benzopyranopyrimidine.^97^ The benzopyranopyrimidine and its derivatives were used for various applications, especially in the medicinal field. The synthesis of this compound requires toxic chemicals, long reaction time, poor yield, and recyclability and reusability of the catalyst were taken into consideration. To avoid this drawback, introducing a heteropoly acid hybrid catalyst would be a better choice. It has a low surface area, an easily soluble nature in organic solvents, and a non-toxic nature. The author introduced the heteropoly acid-supported creation of a functionalized halloysite hybrid catalyst. Owing to halloysite as natural clay, it does not affect the reaction medium, is non-toxic in nature, is inexpensive, and is easily available. The reaction between 4-hydroxycumarin 29 and aromatic aldehyde 30, and diaminoketone 31 presence of an HPA@HNT-C heterogeneous catalyst under ultrasonic irradiation in a water medium provided a benzopyranopyrimidine 32 product with a better yield of up to 95% (Scheme 8). The HPA heterogeneous catalyst provided a better yield at a very short reaction time, and the catalyst was separated easily and reused for another set of reactions. When different substrates were used, better product yields were obtained.

**Scheme 9:**
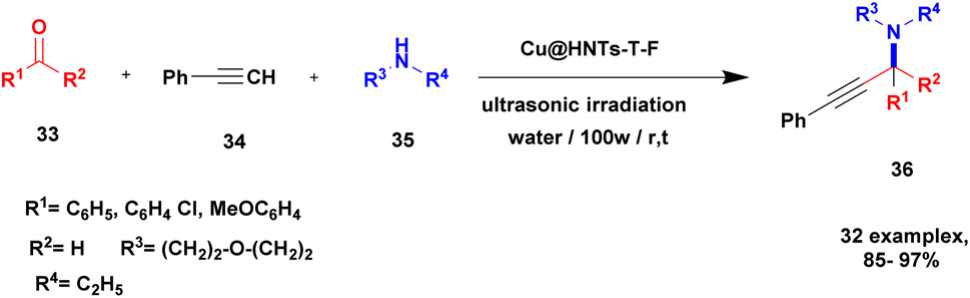
Preparation of propargyl amine derivatives using Cu@HNTs-T-F.

A Cu@furfural imine-supported halloysite clay heterogeneous catalyst was used for three-component A^3^ and KA^2^ coupling reactions to synthesize propargylamine derivatives for various applications.^98^ In the last few years, green chemistry has been prominent owing to the non-toxic and eco-friendly synthesis of compounds to reduce greenhouse gas formation. Halloysite nano clay is an easily available and cheap material that is non-toxic in nature. The surface functionalized halloysite clay was launched recently, and it exhibited excellent activity in various fields for different applications. The author proposed a furfural absorbed copper-supported halloysite nano clay heterogeneous catalyst, and the catalytic performance of the prepared catalyst was studied by performing A^3^ and KA^2^ coupling. The reaction between an aldehyde 33, phenylacetylene 34 and secondary amine 35 in the presence of Cu@HNTs-T-F heterogeneous catalyst and water as the solvent using the ultrasonic method yielded the expected product propargylamine 36 with a better yield. The same reaction was carried out using different catalysts, such as CuI-supported HNT and CuCN with better yield, but the reaction took more time compared to the furfural-supported catalyst (Scheme 9). The different substituted substituents bound to various substrates provided better yields in a shorter time. The simple filtration method was used to separate the catalyst, washed with a (1 : 1) ratio of water and ethanol solution and was reused for another set of reactions. The catalyst was used for four consecutive cycles, which resulted in a better yield without loss in catalytic activity.

**Scheme 10:**
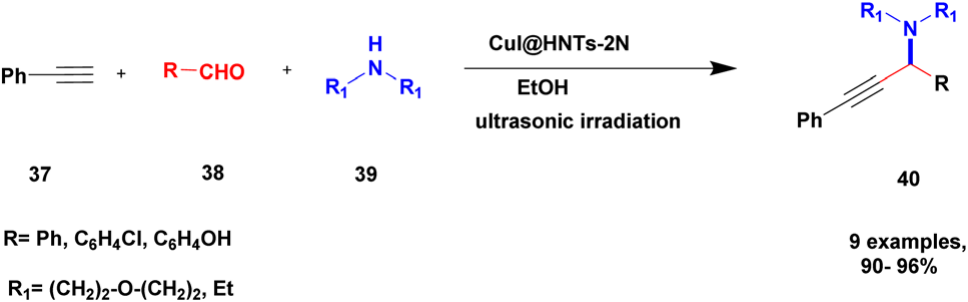
Synthesis of propargylamine derivatives using CuI@HNT-2N catalyst.

Sadjadi and his group^99^ documented a visible light-mediated A^3^ coupling reaction *via* CuI@amine functionalized modified halloysite clay, and a heterogeneous catalyst was used to upgrade the coupling for the preparation of propargyl amine derivatives. In the past decade, many drawbacks of propargyl amine synthesis have been reported. It requires high temperature, a long reaction time, a specific atmosphere and a specific solvent. To avoid these limitations, the authors introduced inexpensive environmentally friendly halloysite clay nanotubes. The functionalized halloysite nanotubes exhibited excellent activity compared with pure HNTs. Here, CuI-supported amine-functionalized HNTs were introduced owing to their outstanding activity, and the CuI@ HNT-2N composite provided a better yield than CuI@ HNTs-N. The reaction was executed between phenylacetylene 37, aldehyde 38 and secondary amine 39 in the presence of copper-supported halloysite clay catalyst CuI@HNTs-N or CuI@HNTs-2N in ultrasonic irradiation (Scheme 10). The two-nitrogen containing catalyst provided propargyl amine 40 products with a better yield than a single nitrogen CuI@ HNT composite with up to 93%. When the reaction was executed in different solvents, ultrasonic irradiation in the ethanol medium provided an excellent yield of 95% compared with the others. The CuI@ HNT-2N catalyst exhibited the best catalytic activity and reduced the reaction time, and the catalyst was regained easily and reused for five cycles without loss of its activity.

**Scheme 11:**
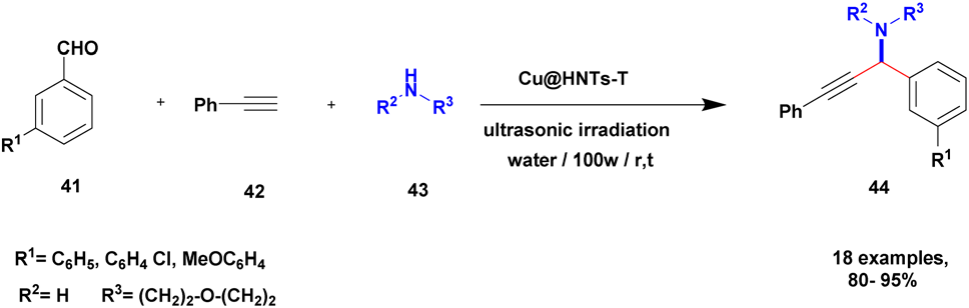
Cu@HNT-T catalyzed A^3^ coupling reaction.

Sadjadi and his group^100^ documented an extension of the above work. The authors prepared copper incorporated a 1,2,4-triazole-5-methanol functionalized halloysite nano clay catalyst. To execute the one-pot three-component A^3^ and KA^2^ coupling reactions for the synthesis of propargylamine derivatives, the reaction was carried out using a conventional method and had some drawbacks. This was avoided by the author by introducing the Cu@HNT-T catalyst. This was driven by the functionalization of HNTs with 1, 2, 4-triazole-5-methanol and then incorporated into copper species. The reaction between carbonyl compounds aldehyde or ketone 41, phenylacetylene 42 and amine 43 in the presence of Cu@HNTs-T catalyst under an ultrasonic medium in a (10 : 1) ratio of water : ethanol solvent system yielded propargylamine 44 was formed with up to 95% yield (Scheme 11).

**Scheme 12:**
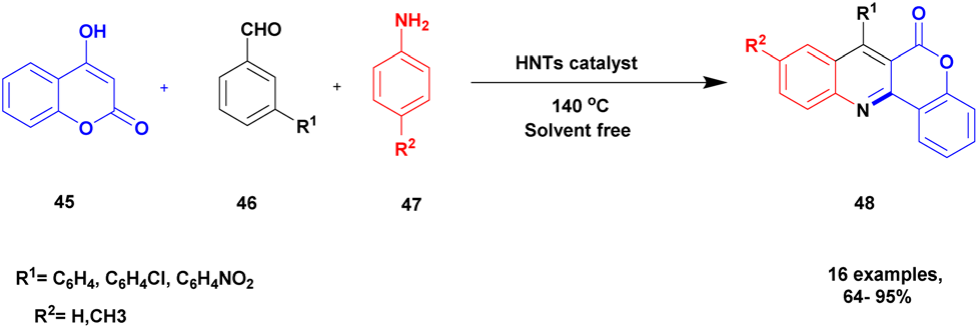
Construction of fluorophore chromeno [4, 3-*b*] quinoline-6-one using HNT-catalyst.

Kachoui and group^101^ documented a protocol for the multicomponent synthesis of fluorophore chromeno [4, 3-*b*] quinoline-6-one using a solvent-free greener method and halloysite nano clay as a catalyst. The chromeno-quinoline compounds exhibited very good medicinal properties used against various infections. The earlier method for the synthesis of the compound requires a high temperature and long reaction time, and some of the solvents used are toxic in nature. To avoid these drawbacks, the authors introduced a natural halloysite clay catalyst to perform the reaction. The halloysite clay is easily available, low cost and non-toxic in nature. It has a high surface area and is kinetically and thermally more stable, and it is used in various fields for different application purposes. The reaction between 4-hydroxy coumarin 45, aromatic aldehyde 46, and *p*-toluidine 47 in the presence of halloysite clay heterogeneous catalyst under the solvent-free condition at 140 °C provided fluorophore chromeno quinoline 48 with a better yield of up to 85% (Scheme 12). When the reaction was carried out in different catalysts and on different substrates, a poor yield was obtained, and when the temperature was decreased or increased above or below 140 °C, a low yield of the product was obtained. The catalyst was reused for five consecutive cycles, which resulted in a better product, and the yield of the product was decreased by 2% after the third cycle, which may be attributed to better catalytic activity.

### 2.3 Hydrotalcite clay-based catalyst using C–N bond formation reactions

**Scheme 13:**
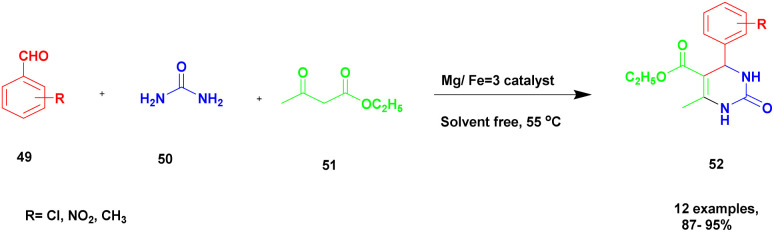
One-pot synthesis of dihydropyrimidinone using Mg/Fe = 3 hydrotalcite catalyst.

Dabholkar and group^102^ addressed a protocol for highly efficient Mg/Fe = 3 hydrotalcite catalysts introduced to execute the Biginelli reaction for the construction of dihydropyrimidones using the one-pot solvent-free synthesis method. Over the past decades, the synthesis procedure had some disadvantages and required harsh reaction conditions, high temperatures, expensive reagents, and poor yield. These drawbacks have been overcome by introducing hydrotalcite clay, which has high surface reactivity. When hydrotalcite is calcinated with Mg/Fe at different molar ratios, the 3 : 1 ratio of calcinated Mg/Fe hydrotalcite exhibits excellent catalytic activity. The basicity of the hydrotalcite steadily increases with the Mg/Fe molar ratio and reaching its peak at Mg/Fe = 3. Hence, the reaction between aromatic aldehyde 49 and diaminoketone 50 provided an iminium ion, and it was further reacted with ethylacetoacetate 51 in the presence of Mg/Fe = 3 hydrotalcite calcinated catalyst using a solvent-free one-pot synthesis method (Scheme 13). These methods provide dihydropyrimidone 52 with an excellent yield of up to 90%. The same reaction carried out in different substituent substrates with electron-deficient or electron-rich groups attached with aromatic aldehyde provided a very good yield. Without calcination, a lower yield was obtained. The major advantage of the calcinated clay catalyst is that it can be easily recovered from the reaction mixture without the loss of its catalytic activity and can be reused for another set of reactions.

**Scheme 14:**
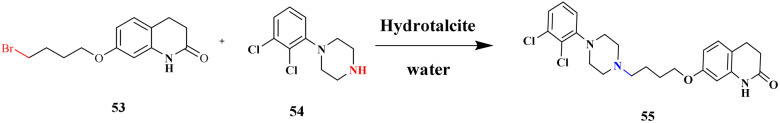
Hydrotalcite catalyzed N-alkylation.

Soni and group^103^ documented a protocol to synthesize N-alkylation products using hydrotacite anionic clay, which is an efficient catalyst. This method is a greener approach to reduce the formation of environmentally affected greenhouse gas without any hazardous chemical components to synthesize the expected product. In past decades, a large amount of chemicals has been used to prepare the expected product, and some of it is toxic in nature, providing a large amount of greenhouse gas and affecting the environment. This drawback has been overcome by the author using a greener method to prepare hydrotalcite clay catalysts for N-alkylation. When hydrotalcite was used, a very small amount of carbon dioxide was produced, which was much better than the earlier methods. The reaction between 7-(4-bromo butoxy)-3,4-dihydroquinoline 2*H*-one 53 with 1-(2,3-dichloro phenyl) piperazine 54 and water in the presence of a lesser amount of hydrotalcite catalyst resulted in the N-alkylation product aripiprazole 55 with an excellent yield of up to 94% (Scheme 14). When similar N-alkylated products were prepared using the hydrotalcite catalyst, a better yield was obtained. When the reaction was completed, the catalyst was regained by filtration and used for another set of reactions. The main advantage of the catalyst was that it reduced the formation of carbon dioxide greenhouse gas and reusability.

**Scheme 15:**
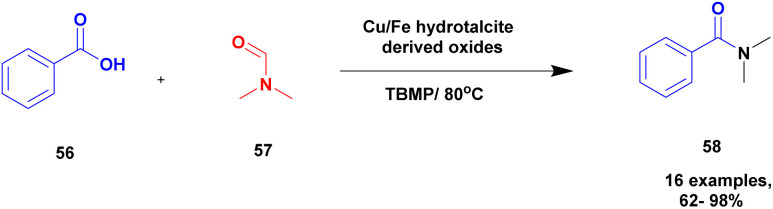
Oxidative coupling of DMF and carboxylic acid.

Priya and group^104^ proposed an oxidative coupling reaction under the calcinated hydrotalcite clay catalyst for the synthesis of *N*, *N*-dimethyl substituted amides. Initially, the oxidative amidation reaction was carried out using different catalytic mediums but had drawbacks, such as expansive chemicals, long reaction time, and the reuse of the catalyst. These limitations were overcome using hydrotalcite clay as the catalyst owing to the specific selectivity and specific activity of product formation. The 3 : 1 ratio of M^2+^/M^3+^ transition metal ions was used for calculations with hydrotalcite oxide formation of the heterojunction catalyst. Cu–Fe calcinated hydrotalcite oxides were introduced, and they exhibited excellent activity compared with other calcinated materials. The oxidative coupling between benzoic acid 56 and DMF 57 in the presence of calcinated Cu/Fe = 3 : 1 hydrotalcite derived oxide catalyst and TBMP occurred by heating the mixture, and the expected *N*, *N*-dimethyl amide 58 product was formed with an excellent yield of up to 92% (Scheme 15). When the same reaction was carried out using different transition metals, calcinated hydrotalcite catalysts with very poor yields were obtained. Additionally, the reaction was carried out using different substituted aromatic acids, and products were formed in low yields. Halogens, such as Cl and Br substituted aromatic acids, provided excellent yields with up to 98%. The advantage of the catalyst was that the reaction was completed at a faster rate, non-hazardous, low cost, easily recoverable and reusability of the catalyst for four cycles without loss of its activity.

### 2.4 Montmorillonite clay-based catalyst using carbon–nitrogen bond formation

#### 2.4.1 Montmorillonite K-10 clay catalysed carbon–nitrogen bond formation

**Scheme 16:**
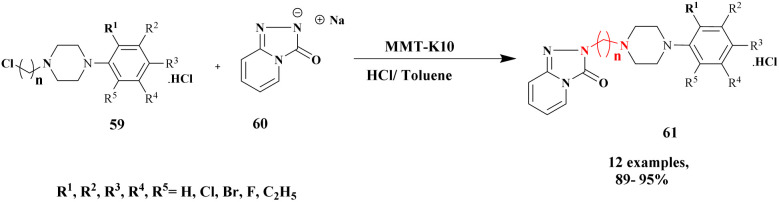
Synthesis of trazodone hydrochloride under a montmorillonite catalyst medium.

Lambat and the group discussed a protocol for the synthesis of trazodone hydrochloride compounds using the greener method with montmorillonite clay heterogeneous catalysts. It has more advantages, such as being non-toxic, easily available, not affecting the environment, easily removable from the catalyst, and reusability. They reported the synthesis of trazodone hydrochloride compounds by applying a greener method presence of a montmorillonite heterogeneous catalyst. Trazodone has promising medicinal properties in the pharmacology field mostly used against various diseases. The reaction between 1-(3-chlorophenyl)-4-(3-chloropropyl) piperazine 59 and 1, 2, 4-triazolo [4, 3-*a*] pyridine-2(*H*) one 60 in the presence of a montmorillonite K10 catalyst with acetonitrile solvent at 90 °C produced trazodone hydrochloride 61 with 90% yield (Scheme 16). When the reaction mixture was cooled at room temperature, it was washed with HCl/toluene mixture, and acetonitrile was removed.^105^ After the reaction was completed, the catalyst was separated by simple filtration without a deficit in its catalytic actitpgoto "vity.

**Scheme 17:**
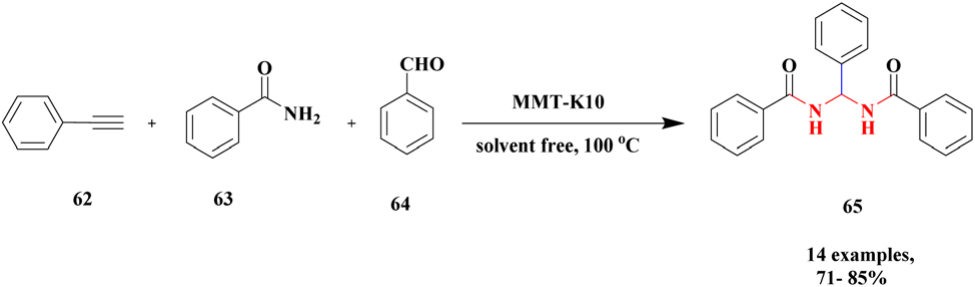
Synthesis of *N*, *N*-alkylidene diamide presence of montmorillonite K10 catalyst.

The synthesis of *N*, *N*-alkylidene bisamide *via* one-pot synthesis under a solvent-free situation in the presence of a montmorillonite K10 heterogeneous catalyst was discussed by Lambat and group.^106^ Initially, many catalysts were introduced to prepare *N*, *N*-alkylidene bisamide, but they had few drawbacks, such as sensitivity to harsh medium, reaction time and multiple byproducts. To avoid these drawbacks, the reaction was carried out under solvent-free greener conditions. Here, the authors performed the reaction with a montmorillonite K10 heterogeneous catalyst, which provided a better yield under solvent-free conditions. Compared to the earlier method, this provided a better yield without any by-product formation. The reaction (Scheme 17) between phenylacetylene 62 and benzamide 63 with benzaldehyde 64 in the presence of MK10 heterogeneous catalyst was heated at 100 °C, and the expected alkylidine bisamide 65 product was formed with a better yield of 85%. Through this protocol, the catalyst was separated from the reaction mixture and reused for another set of reactions, which extended to about four cycles.

**Scheme 18:**
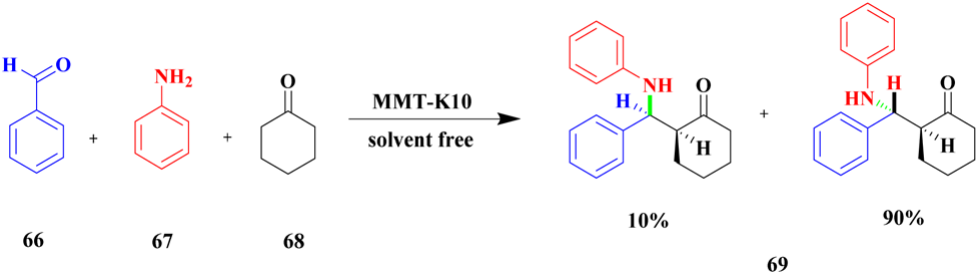
Synthesis of β-amino ketone *via* solvent-free montmorillonite K10 catalyst.

Sanz and group^107^ reported a mannich reaction for the preparation of β-amino ketones using a solvent free one-pot multicomponent synthesis method. The mannich product has very good activity in various fields mainly in the medical field to prepare synthetic drugs against various diseases. The montmorillonite K10 clay overcame the earlier methods and provided a higher yield. The reaction between aromatic aldehyde 66 and aniline 67 with cyclohexanone 68 in the presence of montmorillonite K10 clay catalyst at 38 °C provided a β-amino ketone 69 of 60 : 40 ratio of 3-anti/3-syn product 94% at 120 min (Scheme 18). The same reaction was carried out at 5 h with a montmorillonite K10 catalyst, and a selective 3-anti product was formed with 90% yield.

**Scheme 19:**
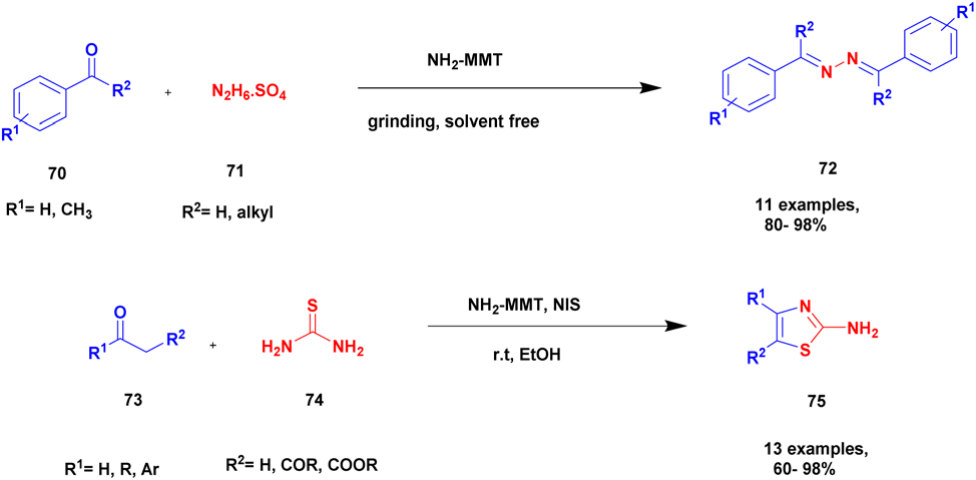
Synthesis of azines and 2-amino thiazole using NH_2_-MMT catalyst.

Zarnegar and group^108^ addressed the synthesis of azines and 2-aminothiazoles using a solvent-free greener method with amine-modified montmorillonite clay as a heterogeneous catalyst. Both azine and 2-aminothiazole derivatives have diverse biological activities and are used in the medicinal field for various applications. However, the earlier methods used in the preparation of the compounds have a few drawbacks, such as prolonged reaction time, poor product formation, hazardous chemicals, many catalysts and reagents needed, and require very high temperatures. To avoid these limitations, a reaction is executed using the solvent-free greener method with a green catalyst. The authors introduced an amine-modified montmorillonite clay (NH_2_-MMT) heterogeneous catalyst, which does not affect nature and the environment. This amine-modified MMT exhibits both acidic and basic properties. The reaction between aldehyde 70 and hydrazine sulfate 71 in the presence of NH_2_-MMT catalyst using a solvent-free grinding method provides an excellent coupling product azine 72 with a better yield. Next, methyl carbonyl 73 was reacted with thiourea 74 and *N*-iodosuccinimide in the presence of ethanol and NH_2_-MMT catalyst, and a good coupling product 2-aminothiazole 75 was observed with a better yield (Scheme 19). The azines can be prepared using different percentages of NH_2_-MMT catalyst both in solvent-free grinding method and reflux method, and the solvent-free grinding method provides a very good to a better yield of up to 94% yield. The 2-aminothiazole was prepared using various solvent mediums with different iodine precursors, but the *N*-iodosuccinimide precursor in the presence of ethanol medium with NH_2_-MMT catalyst provided an excellent yield of up to 97% yield.

**Scheme 20:**
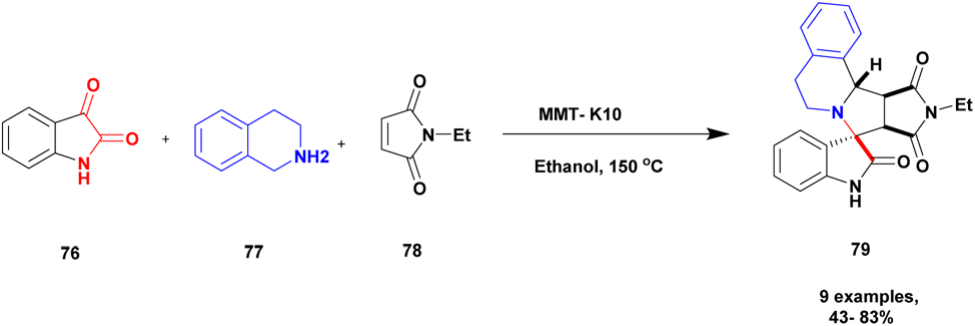
Synthesis of spiro oxindole under a montmorillonite K10 clay catalyst.

Zhang and his group^109^ addressed a protocol for the synthesis of spiro oxindole *via* one-pot synthesis with a montmorillonite K10 clay heterogeneous catalyst to perform the reaction. The spiro oxindole has various biological activities. Hence, the author carried out a [3 + 2] cycloaddition reaction using a greener method in the presence of a montmorillonite K10 clay catalyst. Montmorillonite K10 is easily available, cheap, environmentally friendly and easily recoverable from the reaction mixture and reusable. The reaction between isatin 76 and 1, 2, 3, 4-tetrahydroisoquinoline 77 and *N*-ethylmalemide 78, in the presence of ethanol solvent and montmorillonite K10 clay heterogeneous catalyst at 150 °C [3 + 2] cycloaddition reaction occurred to provide spirooxindoles 79 better yield with up to 80% (Scheme 20). Initially, the nucleophilic addition of tetrahydroisoquinoline to isatin and then the dehydration process occurred, and an iminium ion was formed. Then, the iminium ion converted into azomethine after deprotonation [3 + 2] cycloaddition occurred with maleimide; finally, the expected product spiro oxindole was formed.

**Scheme 21:**
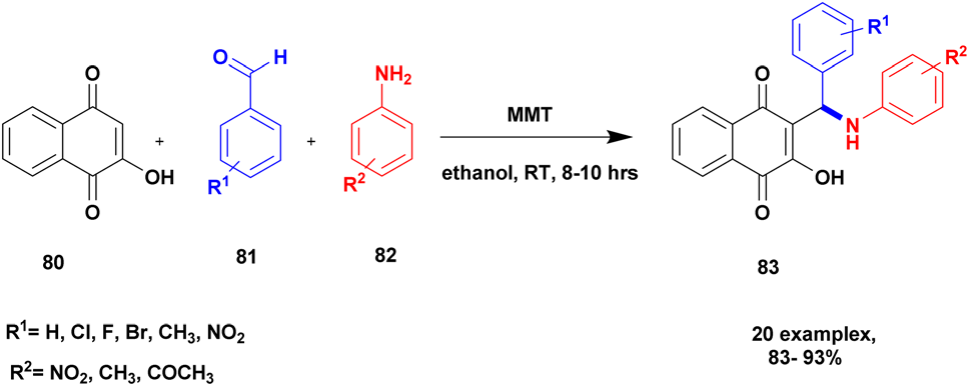
Synthesis of napthoquinoline with montmorillonite K10 catalyst.

Jayashree and group^110^ reported a protocol for the synthesis of aminonaphthoquinone derivatives with montmorillonite clay K10 heterogeneous catalysts. The napthoquinoline is mostly used in the medicinal field to cure health problems. In the previous method, the compound was prepared with a transition metal, and a transition metal complex catalyst was used as a catalyst. This method has many drawbacks: it is high cost, toxic in nature, and cannot recycle the catalyst easily. To avoid these limitations, the author introduced a natural montmorillonite K10 clay catalyst, which is easily available, non-toxic, easily separated from the reaction mixture and reusable. The reaction between 2-hydroxy-1,4-napthoquinone 80 and benzaldehyde 81 with aniline 82 in the presence of a montmorillonite K10 catalyst and ethanol solvent provides amino napthaquinoline 83 with a better yield of up to 93% (Scheme 21). When the reactions were performed using various solvents and catalysts, they yielded poor products.

**Scheme 22:**
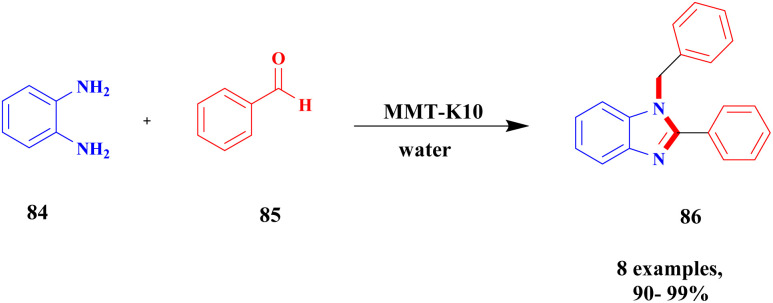
Synthesis of propargylamine derivatives using the greener method.

Bonacci *et al.*^111^ addressed a procedure for the preparation of benzimidazole derivatives using a montmorillonite K10 heterogeneous catalyst. Benzimidazole has numerous biological applications, and the earlier method of synthesis of the compound has some drawbacks. They needed expensive and toxic solvents and a long reaction time, and recycling of the catalyst was a very tedious process. The heterogeneous catalyst was developed in the last few decades, and it has excellent activity and more applications in various fields because it is more stable and easily recoverable. The authors performed the reaction under microwave irradiation with a natural montmorillonite K10 clay heterogeneous catalyst. Owing to the montmorillonite K-10 features, such as being easily available, non-toxic in nature, and easily recoverable from the reaction mixture, microwave irradiation increases the rate of the reaction and decreases the waste by-product formation. The reaction between *ortho*-phenylenediamine 84 and aromatic aldehyde 85 in the presence of montmorillonite K10 heterogeneous catalyst with water as a solvent benzimidazole 86 derivative was formed with a better yield (Scheme 22). When performed, the reaction with different substrate selective products was obtained. MK10 helps to reduce waste product formation.

**Scheme 23:**
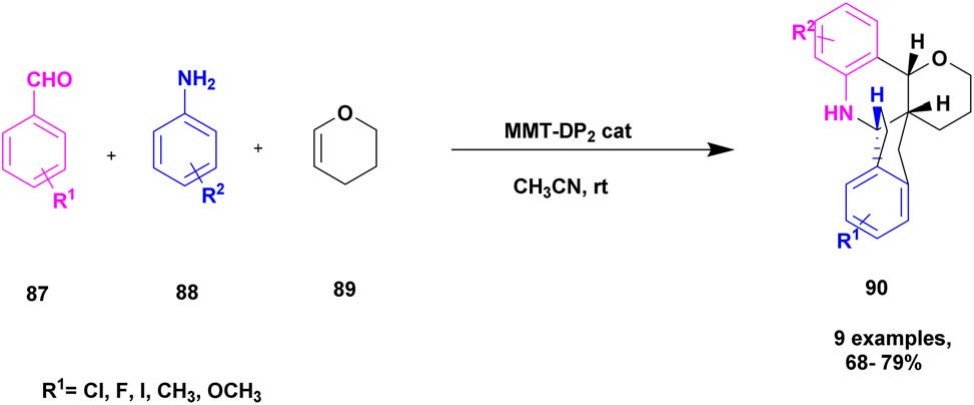
Synthesis of pyranoquinoline *via* a Mont-K10 clay-supported DP_3_ Schiff base metal complex catalyst.

Kannan and group^112^ reported a montmorillonite K10-supported metal, and a Schiff base complex was used to synthesize pyranoquinoline under aza-Diel's alder reaction. The asymmetric synthesis was an important tool for the construction of novel optically active compounds with very good biological activity. In the past decades, the synthesis of these optically active compounds was carried out using the aza-Diels–Alder reaction in the presence of an excellent asymmetric catalyst. The authors introduced a montmorillonite K10 clay-supported peptide metal complex to prepare optically active compounds. The reaction was carried out without MMT-K10, and it was difficult to remove the Schiff base from the reaction mixture. Mont-K10-supported Cu and Ti metal complexes were used to perform an aza-Diels–alder reaction with a better yield, and MMT-K10 helped to reduce the Schiff-base formation (Scheme 23). The reaction between aromatic aldehyde 87, aniline 88 and 3, 4-dihydro 2*H*-pyrene 89 in the presence of mont-K10-supported dipeptide Schiff base metal complex provides fused quinoline 90 with a better yield of 84%.

**Scheme 24:**
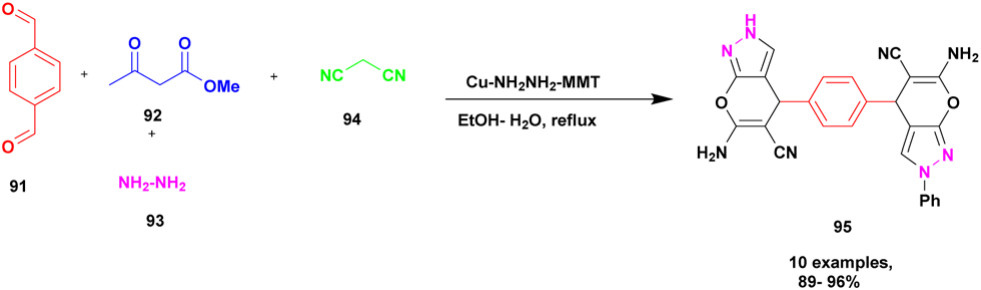
Synthesis of bispyrano[2,3-*c*] pyrazole using Cu–NH_2_–NH_2_-MMT catalyst.

Synthesis of bispyrano [2,3-*c*] pyrazole was achieved by Ahmadzadeh *et al.*^113^ using the one-pot multicomponent method in the presence of copper incorporated amine modified montmorillonite clay catalyst. The four-component coupling occurring between aromatic aldehyde 91, ethyl acetoacetate 92, hydrazine 93, and malononitrile 94 in the presence of Cu (ii) anchored amine-modified MMT clay catalyst provided the expected bispyrano [2,3-*c*] pyrazole 95 compounds of up to 95% within 15 min under water–ethanol solvent medium (Scheme 24). The first condensation reaction occurred between aldehyde and malononitrile, which generated one intermediate; simultaneously, condensation occurred between ethylacetoacetate and hydrazine. Finally, both intermediates are cyclized to form the coupled product. Even for four cycles, the catalyst was reusable, and no catalytic changes were observed. The advantages of this catalyst were its reusability and short reaction time.

**Scheme 25:**
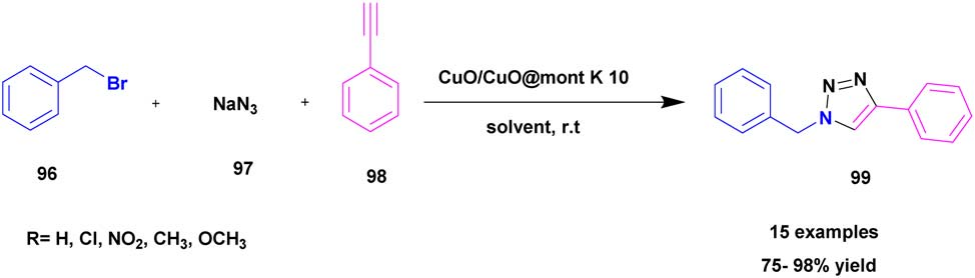
Synthesis of 1,2,3-triazoles using Cu_2_O/CuO@MK-10.

Pathak and his research group^114^ published a work of azide–alkyne cycloaddition reaction for the synthesis of 1,2,3-triazoles 99 using a Cu_2_O/CuO@MK-10 heterogeneous catalyst. A simple one-pot click chemistry method was used for synthesizing 1,2,3-triazole. The reaction between sodium azide 97 and phenyl acetylene 98 with benzyl halide 96 in the presence of Cu_2_O/CuO@MK-10 (10 mg) in water solvent medium at room temperature gave 98% yield in 1 h. The reaction was optimized by changing the catalyst (MK-10, Cu_2_O, CuO, Cu_2_O/CuO@MK-10 (5 mg, 10, 20 mg) and solvent (water, DCM, ethanol, 1 : 1 ethanol : water). The optimization in the presence of Cu_2_O/CuO@MK-10 (10 mg) catalyst in a water solvent medium gave a good yield (Scheme 25). The reaction carried out with different substituent attached derivatives gave a 75–98% yield. When the electron donating group CH_3_, OCH_3_ and unsubstituted compound gave 85–98% yield, the electron withdrawing groups Cl and NO_2_ gave 85–90% yield. The catalyst was separated using a simple filtration technique, then washed with ethanol and reused for another reaction. The catalyst recycled and reused even for five cycles exhibited good catalytic activity.

**Scheme 26:**
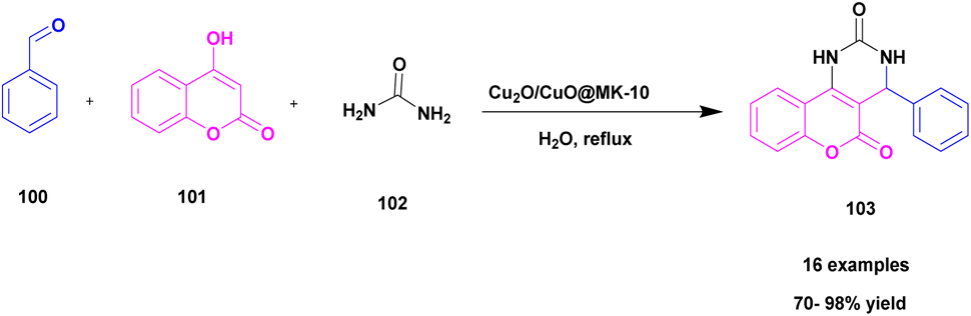
Synthesis of benzopyranopyrimidines derivatives using (Cu_2_O@Mont/EAS-1L).

Besharathi and his research group^115^ developed a protocol for the synthesis of benzopyranopyrimidine derivatives 103 using Cu_2_O immobilized MK-10 decorated ionic-liquid catalyst (Cu_2_O@Mont/EAS-1L). The reaction occurred between aromatic aldehyde 100, 4-hydroxy coumarin 101 and urea/thio-urea/guanidine 102 in a water solvent medium in the presence of Cu_2_O@Mont/EAS-1L catalyst at 60 °C gave a good yield (70–98%) within 15 min. The reaction was optimized under different conditions of catalyst concentration (15, 25, and 35 mg), solvents (water, ethanol, DMF, chloroform, and acetonitrile) and temperature (r.t, 60 °C, and reflux). Finally, the optimization presence of 25 mg Cu_2_O@Mont/EAS-1L catalyst in water solvent medium at 60 °C showed good activity with 70–98% yield. The reaction was carried out with different substituent attached derivatives, and both electron donating and electron withdrawing groups gave 70–86% yield. When compared with the electron donating & electron withdrawing groups, the unsubstituted derivative gave a good yield of 95–98%. Additionally, compared with earlier reported catalysts, the Cu_2_O@Mont/EAS-1L showed good catalytic activity with 98% yield. The catalyst was easily recycled from the reaction mixture by applying the normal filtration technique and reused for another set of reactions. After 5 times of recycling, the reactions showed good catalytic activity with 82% yield (Scheme 26).

**Scheme 27:**
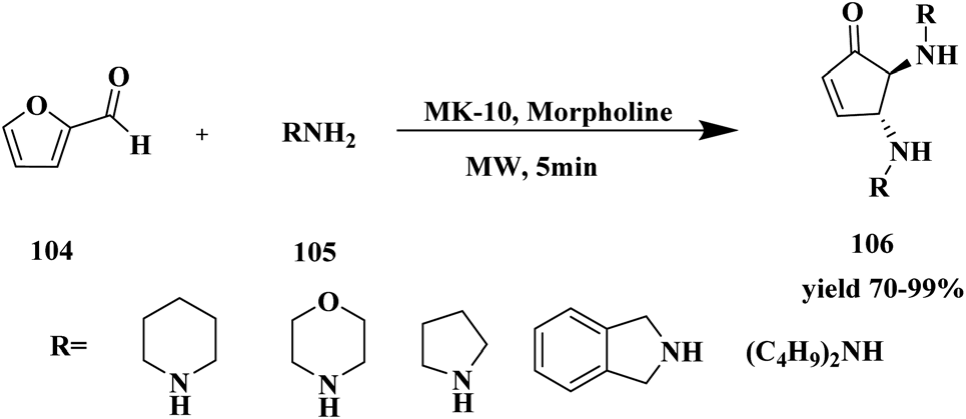
Synthesis of bi-functionalized cyclopentanones using MK-10.

Bonacci *et al.*^116^ developed a protocol for the synthesis of bi-functionalized cyclopentanones 106*via* solvent free micro-wave medium using montmorillonite K-10 catalyst. The functionalized cyclopentanones were synthesized from furfural, and it was one of the starting materials. Furfural-based compounds are one of the building blacks for the synthesis of various bio active compounds. Nowadays, numerous furfural-based compounds are used as biologically active compounds. From the chiral difunctionalized cyclopentanones, the *trans*-4,5-disubstituted cyclopentenones exhibited good activity. The reaction carried out between 1 mmol of furfural 104 and 2 mmol of amine 105 in the presence of 20 mol% of MK-10 catalyst at 60 °C for 5 min in microwave showed 99% conversion with 98% yield. The reaction was optimized with different weight percentages of catalyst (10%, 20%, without catalyst), and temperature (r.t, 60 °C, 80 °C, and 100 °C). Additionally, the reaction was carried out with different substituted derivatives (Scheme 27). Finally, the optimization in the presence of MK10–20% at 60 °C in MW showed a 99% yield of *trans*-4,5-dimorpholinocyclopent-2-enone within 5 min. The catalyst recycled and reused for the further synthesis of bi-functionalized cyclopentanones showed good catalytic activity and high conversion with good yield after the third cycle.

#### 2.4.2 Montmorillonite-KSF clay-mediated carbon–nitrogen bond formation

**Scheme 28:**
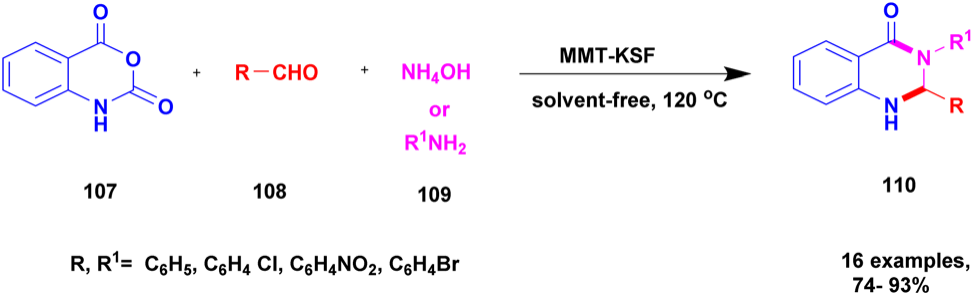
Synthesis of 2, 3-dihydroquinazolineone using an MMT-KSF clay catalyst.

Tekale *et al.*^117^ addressed a protocol for the synthesis of 2, 3-dihydroquinazoline 4-1(*H*) one using the solvent-free method with a montmorillonite-KSF clay heterogeneous catalyst. The quinazoline compounds possess excellent biological activity for the treatment of various diseases, but the earlier method of the synthesis of the compound has some drawbacks. To overcome the disadvantages of the past decades, the authors used a greener method and carried out the reaction using montmorillonite-KSF clay. It is an easily available one and should not affect the nature and environment. The reaction between isatoic anhydride 107 and aldehyde 108 with ammonium acetate or amine 109 in the presence of MMT-KSF (10% wt) heterogeneous catalyst using solvent-free microwave method provided the expected product 2, 3-dihydroquinazoline 110 with a better yield of up to 87%. Then, different catalysts and solvents used to carry out the same reaction with different substrates using MMT-KSF (10% wt) provided a better yield (Scheme 28). The merits of using the MMT-KSF (10% wt) catalyst were that the reaction was completed in a short time compared with an earlier method, the catalyst was easily recovered and reused for another reaction, and it was non-hazardous and did not affect the environment.

**Scheme 29:**
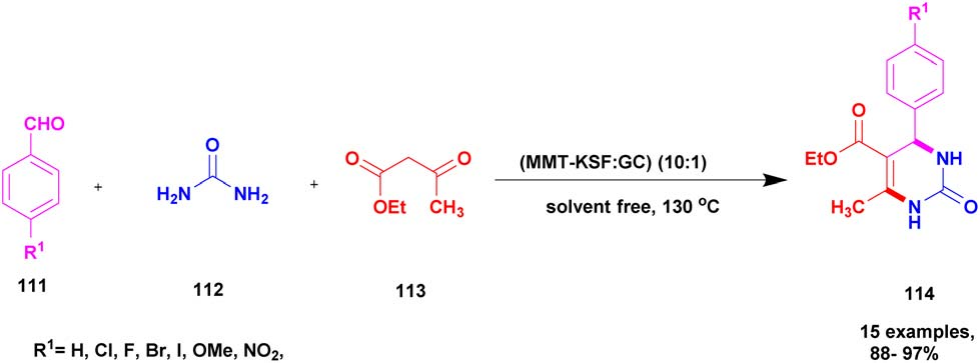
Synthesis of 2, 3 dihydropyrimidinone using CG (10 : 1) catalyst.

Narayanan and group^118^ reported a protocol to carry out the Biginelli reaction for the synthesis of dihydropyrimidinone using the solvent-free multicomponent method with montmorillonite-KSF clay – graphene oxide used as a heterogeneous catalyst. The Biginelli type of compound exhibits excellent biological activity and is used against various diseases and infections. Initially, the Biginelli derivatives are prepared using various catalysts, both heterogeneous and homogeneous catalysts. They have some drawbacks: poor product formation, toxic-chemical needed to carry out the reaction with expensive reagents, and catalysts required of the synthetic procedures affect the environment. To overcome these limitations, the reaction was performed using the greener method. The authors were the first to report montmorillonite clay–graphene oxide nanocatalysts using multicomponent reactions for the synthesis of Biginelli products. The graphene oxide exhibits excellent thermal and electrical conductivities, and it possesses various applications in various fields in current research. The different percentages of GO-loaded MMT-KSF clay catalysts were investigated for the synthesis of Biginelli products under solvent-free conditions. The MMT-KSF clay was easily available, did not affect the reaction medium and was non-toxic in nature. The reaction between an aromatic aldehyde 111 and diaminoketone 112 with ethylacetoacetate 113 in the presence of mont-KSF-GO clay at a (10 : 1) ratio as the catalyst under solvent-free condition at 130 °C provided 3,4-dihydropyrimidinones 114 in excellent yields of up to 94% (Scheme 29). The reaction carried out in different substrates also provided a better yield, could easily separate the catalyst and be reused even for eight cycles, and has excellent activity with better yield without decreasing its catalytic performance.

**Scheme 30:**
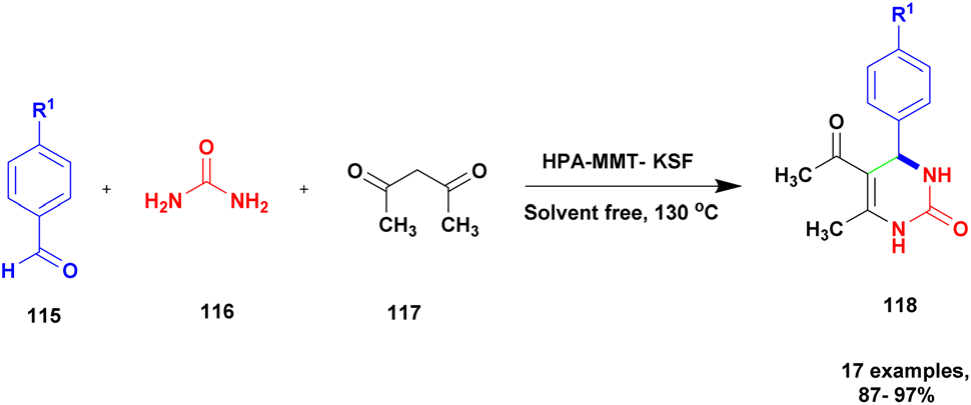
Synthesis of dihydropyrimidinones *via* HPA-montmorillonite catalyst.

Farooq *et al.*^119^ reported a protocol for the construction of dihydropyrimidinones *via* a solvent free one-pot synthesis using heteropoly acid supported montmorillonite KSF clay as a heterogeneous catalyst. Over the last few decades, the unbelievable growth of multicomponent reactions has been observed in organic synthesis. Selective product formation should not require any separation technique for the removal of intermediate compounds and by-products. In previous procedures, intermediates were produced in each stage and purified for use in the following steps. But MCR uses minimal usage of solvent and avoids multi-step synthesis. Dihydropyrimidinone was prepared using the MCR method with a heteropoly acid-supported montmorillonite KSF catalyst. The reaction between benzaldehyde 115 and urea 116 with ethyl acetoacetate 117 provides a dihydropyrimidinone 118 excellent yield with up to 96% yield (Scheme 30). Whether the reaction was performed without clay or HPA, low yields of 82% and 80%, respectively, were obtained; when both clay and HPA were combined, the yield gradually increased up to 96%. The merits of the catalyst were quick reaction time, excellent yield, ease of separation of the catalyst from the reaction mixture without loss of its catalytic activity and reusability for another set of reactions. Additionally, a non-toxic nature should not affect the nature and environment.

**Scheme 31:**
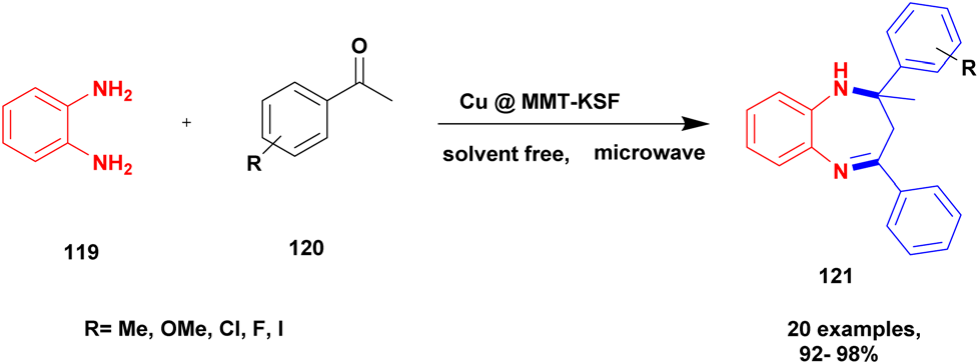
Synthesis of benzodiazephine using Cu@ MMT-KSF clay catalyst.

Shaikh *et al.*^120^ addressed a protocol for the synthesis of 1, 5-benzodiazepines using a solvent-free microwave method with Cu (ii)-doped montmorillonite-KSF nano clay used as a heterogeneous catalyst. The benzodiazepines are used in various fields, such as medicinal, agrochemical, cosmetics and dye and pigment industries. Different types of acidic reagents and iodine molecule-based catalysts as well as some solid phase catalysts have been used in the past decades for the synthesis of benzodiazepine derivatives. They have some disadvantages: poor yield, require high temperature and expensive chemicals to perform the reaction and they affect the environment. To avoid these limitations, the reaction was carried out in an eco-friendly and solvent-free greener method using montmorillonite-KSF clay because it is a naturally available and cheap material. Functionalized montmorillonite clay exhibits excellent activity. Here, the author introduced Cu (ii)-doped MMT clay to synthesize benzodiazephines. The reaction between acetophenone 119 and *O*-phenylene diamine 120 in the presence of Cu (ii)-doped MMT-KSF clay using a solvent-free microwave method provided benzodiazepines 121 in excellent yields of up to 98% (Scheme 31). When the same reaction was performed in the presence of different clay catalysts and different solvent mediums, products were formed in poor yields. Simple separation protocols can easily isolate the catalyst from the reaction medium, which can be used again for another set of reactions. Even after five times, the reused catalyst does not show any significant change and provides an excellent yield. The merits of the catalyst were a short reaction time, recyclability, no need for any tedious process, and no by-product formation occurred.

#### 2.4.3 Heretopolyacid-supported montmorillonite catalyzed carbon–nitrogen bond formation

**Scheme 32:**
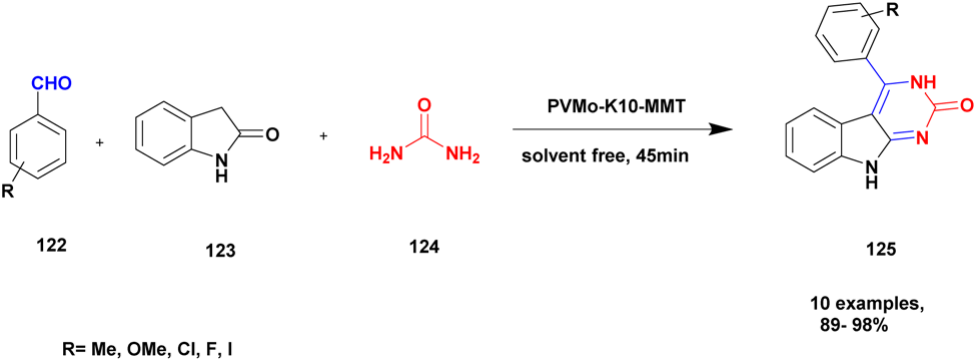
Synthesis of pyrimido [4, 5- *b*] indole using PVMo-K10 catalyst.

The synthesis of pyrimido [4, 5-] indoles using the solvent-free method with a heteropoly-11-molybdo-1-vanadophosphoric acid supported montmorillonite K10 clay catalyst was reported by Kumaresan and group.^121^ The one-pot synthesis method approach provides a high yield compared with the conventional method. The authors executed a reaction between an aromatic aldehyde 122 and oxindole 123 with diaminoketone 124 in the presence of PVMo-supported montmorillonite K10 clay catalyst under the solvent-free method provided pyrimodo indole 125 with a better yield of up to 95% (Scheme 32). The PVMo played a key role in product formation, and without PVMo, low yield was observed. Similarly, the montmorillonite helped to speed up the reaction rate for selective product formation.

**Scheme 33:**
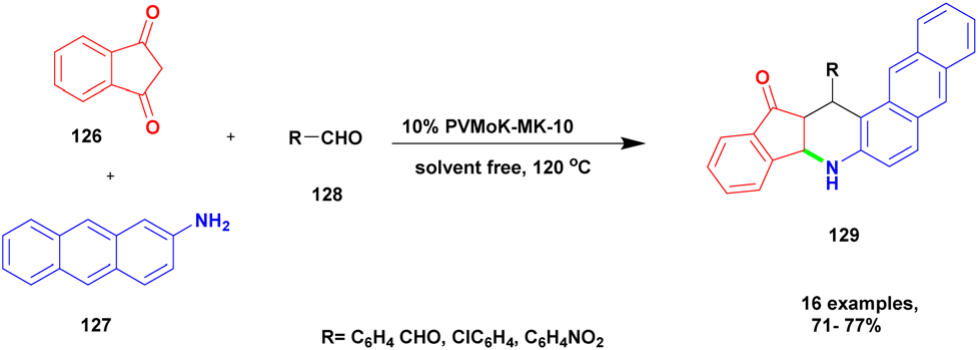
Synthesis of naptho [2, 3-*a*] quinoline using a PVMoK-10 catalyst.

Kumaresan and group^122^ reported a heteropolyacid-assisted montmorillonite K10 dissimilar catalyst for the preparation of naptho [2, 3-*f*] quinoline-13 one and naptho [2, 3, -*a*] acridine 1-2(*H*) one using the solvent-free greener method. Naturally, the quinoline and acridine compounds have diverse medicinal applications, and some of these derivatives are used against various diseases and infections, such as anti-malarial and anti-hypertensive agents. In earlier methods, synthesis of the quinoline and acridine derivatives has some disadvantages, such as extensive reaction time, poor yield, few reactions needing a toxic solvent, and recovery of the metal catalyst from the reaction mixture requires a very tedious process. To avoid these limitations for carrying out the reaction, a greener solvent-free one-pot synthesis method was used, and provides a better yield without any by-product formation. The authors introduced a heteropoly vanadophophoric acid supported montmorillonite K10 clay heterogeneous catalyst for the reaction between 1, 3-indanedione 126 and 2-amino anthracene 127 with aromatic aldehyde 128 and provided the expected naptho [2, 3-*f*] quinoline-13 one 129 with a better yield (Scheme 33). Instead of 1, 3-indanedione, 1, 3-cyclohexadiene was used, and it provided naptho [2, 3-*a*] acridine-1-2(*H*) product. When compared with the other methods of synthesis, product was formed in low yields. The major advantage of the catalyst was the simple method of synthesising the product, and the non-hazardous greener method was used to carry out the reaction.

**Scheme 34:**
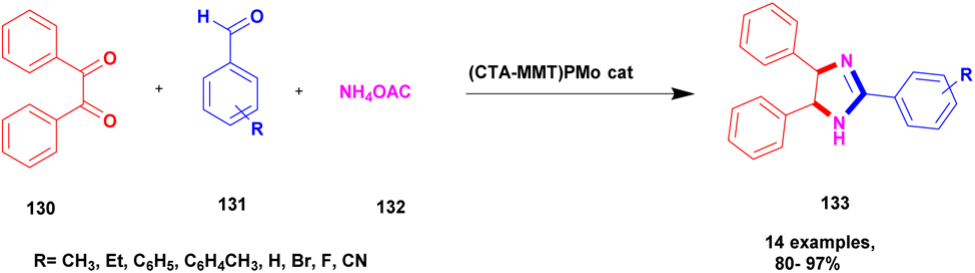
Synthesis of 2, 4, 5-trisubstituted imidazole presence of the (CTA-MONT) PMo catalyst.

Farahani *et al.*^123^ reported the synthesis of 2, 4, 5-tri substituted imidazole using the greener method with montmorillonite clay supported heteropoly acid heterogeneous nanocomposite catalyst. The imidazole derivative compounds exhibited excellent activity, especially in the medicinal field. Previously reported synthesis methods have some drawbacks, and to avoid these drawbacks, the reactions were carried out using a greener method. Phosphomolybdic acid was used in various organic reactions. It provided a very good yield, but one of its drawbacks was the difficulty in separating from the reaction mixture. To avoid these limitations, the authors introduced montmorillonite clay; it has a high surface area and is easily available, and it should arrest phosphomolybdic acid mobilization. For the first time, the authors introduced CTA-montmorillonite clay supported HPA for the preparation of substituted imidazole because it provided a better yield in a short time. The reaction between 1, 2-diketone 130 and aldehyde 131 with ammonium acetate 132 in the presence of clay (CTA-montmorillonite clay) supported HPA catalyst provided tri-substituted imidazole 133 product with better yield (Scheme 34). When the reaction was carried out on different substituted substrates, it also provided a better yield. The major advantage of the catalyst was the greener method of synthesis, which can be easily recovered from the catalyst and reused for another set of reactions.

**Scheme 35:**
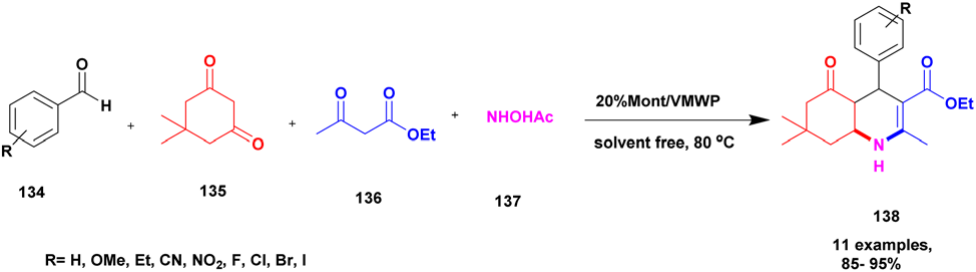
Synthesis of poly hydro quinoline using 20% Mont/VMWP.

Aher *et al.*^124^ reported a Keggin-type phosphoric acid-supported commercial montmorillonite clay heterogeneous catalyst for the preparation of poly-hydro quinoline derivatives. Initially, heteropoly acid was used because it exhibits excellent activity, but it has some demerits, such as poor heat resistance and the least surface area separation from the reaction mixture required a tedious process. To avoid these drawbacks, the authors introduced mixed HPA-keggin type derivatives, such as vanado-molybdotungstophosphoricacid, and subsequently prepared a compound incorporated into natural montmorillonite clay. It has a large surface area, so it helps to increase the catalytic activity of keggin-HPA. The catalytic performance of the prepared catalyst was studied using the preparation of quinoline derivatives. The reaction takes place among benzaldehyde 134, dimedone 135 and acetoacetic ester 136 with ammonium acetate 137, and condensation takes place without solvent at 80 °C, providing polyhydroquinoline 138 at better yield (Scheme 35). When compared with the solvent used method and the presence of any other catalyst, this method at 20% VMWP/Mont provided a better yield with a high percentage. The catalyst was easily recovered by simple filtration and reused for another reaction. Even after four cycles, they provided a better yield without loss of its catalytic activity.

**Scheme 36:**
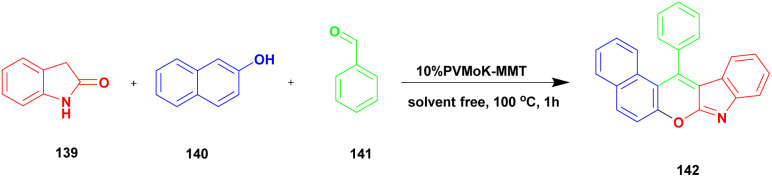
Synthesis of chromeno[2,3-*b*] indoles under 10%PVMoK-MMT catalyst.

Muthu and group^125^ published a one-pot multicomponent synthesis of chromeno[2,3-*b*] indoles under 10%PVMoK (keggin-type of heteropoly-11-molybdo-1-vanadophosphoric acid) supported montmorillonite K-10 clay catalyst. In this reaction, a one-pot three-component condensation reaction occurred between oxindole 139 and β-napthol 140 with aldehyde 141 in the presence of 10% of PVMoK-10 catalyst at 100 °C, providing a coupled chromeno product 142. The authors prepared different % loaded catalysts, among which 10% PVMoK incorporated MMT has better catalytic activity. The mechanistic pathway of the reaction is the first condensation reaction occurring between naphthol and oxindole; subsequently, the condensation product reacts with aldehyde, followed by cyclisation (Scheme 36). Finally, a coupled [2,3-*b*] indole product was obtained. Easily separable, reusable, and eco-friendly materials are the major advantages of the 10%PVMoK-MMT catalyst.

**Scheme 37:**
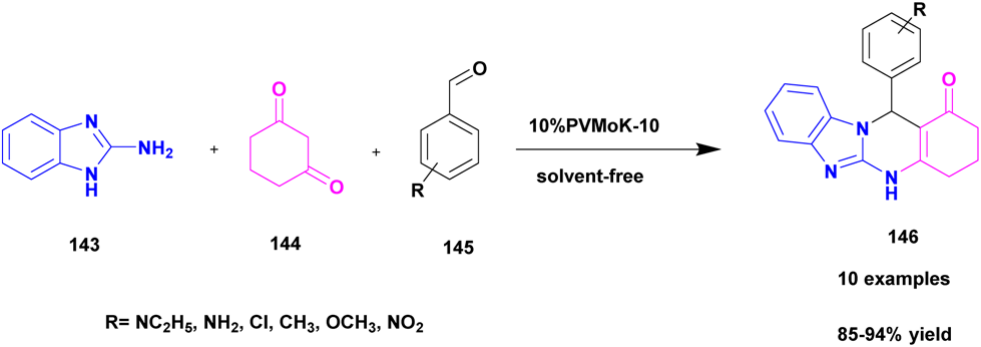
Synthesis of benzoimidazoquinazolinone and indolylxanthenone using PVMoK-10.

Prasanna and his group^126^ in 2022 reported a green approach for synthesizing benzoimidazoquinazolinone and indolylxanthenone derivatives using montmorillonite K-10 clay as a catalyst and keggin-type heteropoly-11-molybdo-1-vanadophosphoric acid. A simple one-pot three-component condensation reaction was used in the protocol, with 10% heteropoly-11-molybdo-1-vanadophosphoric acid (H_4_[PVMo_11_O_40_])-loaded montmorillonite K-10 clay material (PVMoK-10) serving as an efficient heterogeneous catalyst. The overall reaction procedure for the synthesis of benzo[4,5]imidazo[2,1*b*]quinazolin-1(2*H*)-one derivatives 146 is as follows: 2-aminobenzimidazole 143, 1,3-cyclohexadione 144, substituted aromatic aldehyde 145, and 0.05 g of the catalyst 10% PVMoK-10 were heated for one hour at 100 °C (Scheme 37). The performance of 10% catalysts PVMoK-10 and PV_2_Mo-K10 is significantly higher than that of raw mont-K10 clay and vanadium-substituted heteropoly acids. The reactions were carried out in various solvent media, including EtOH, MeOH, H_2_O, MeCN, DCE, DMF, CHCl_3_, 1,4-dioxane, *n*-hexane, and toluene. The results demonstrated that the solvent-free reaction setting was the best for the current synthetic transformation, yielding excellent products. Ten benzimidazoquinazolinone derivatives and two indolylxanthenone derivatives were synthesized with a focus on an environmentally friendly method. This methodology has several advantages, including a short reaction time, high yield, reusability of the catalytic material, a straightforward reaction procedure, and solvent-free reaction conditions.

#### 2.4.4 Na-MMT catalyzed carbon–nitrogen bond formation

**Scheme 38:**
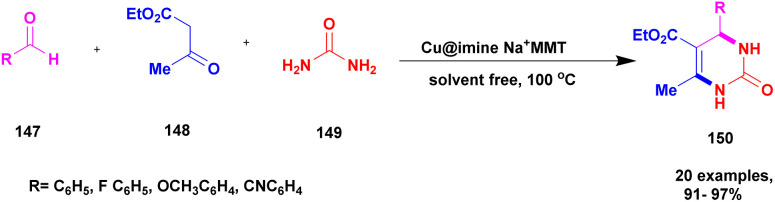
Preparation of 3, 4-dihydropyrimine derivatives using Cu@imineNa^+^MMT catalyst.

Khorshidi *et al.*^127^ addressed a protocol for the preparation of 3,4-dihydro pyrimidine without solvent using a one-pot synthesis method with Cu imine functionalized Na^+^ montmorillonite as a heterogeneous catalyst. The compound 3, 4-dihydro pyrimidine has excellent biological activity against various diseases. In past decades, the synthesis of the compound using the one-pot synthesis method with a metal catalyst has some drawbacks. To avoid these drawbacks, the reaction conducted using the solvent-free greener method provided a better yield. It avoids the formation of a toxic substance and also reduces excess solvent and waste byproduct formation. The authors introduced Na^+^-montmorillonite clay catalyst with and without Cu functionalization; the Cu-functionalized catalyst exhibited excellent activity and a low product yield was obtained using the catalyst without Cu functionalization. Cu@imineNa^+^-montmorillonite, the functionalized compound, exhibited excellent catalytic activity with the expected product. The reaction between aldehyde 147, ethylacetoacetate 148 and diaminoketone 149 in the presence of Cu@imineNa^+^-mont heterogeneous clay catalyst under the solvent-free condition at 100 °C provided dihydropyrimidine-one 150 with a better yield (Scheme 38). When the reaction was carried out with different substituted substrates, a better product was formed. In addition, a catalyst can be easily separated using a simple filtration method and then washed and reused for another set of reactions, providing a better product after the fifth cycle without loss of its catalytic performance.

**Scheme 39:**
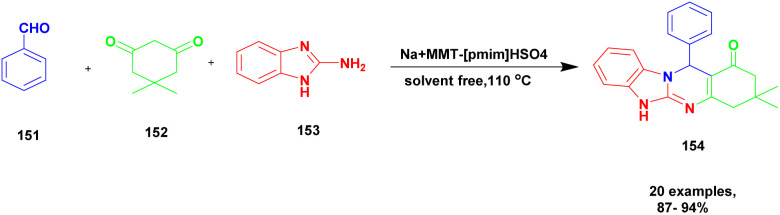
Synthesis of benzoimido quinazoline using Na^+^ MMT [pmim] HSO_4_ catalyst.

Shirini *et al.*^128^ reported a protocol for the synthesis of tetrahydrobenzimidazo [2, 1-*b*] quinazoline-1(2*H*)-one *via* solvent-free conditions using sodium montmorillonite clay incorporated with a Brønsted acidic ionic liquid as the catalyst. The benzimidazo quinazolinones were key compounds for the synthesis of the various biologically active compounds. In recent decades, synthetic methods have had some demerits. For example, they require expensive solvents and catalysts and a hazardous chemical, and some reactions need metal catalysts to proceed. Additionally, a very tedious process occurred that separated the catalyst from the reaction mixture. To avoid this limitation, the authors performed the reaction using the green chemistry method. The three Na^+^ montorillonote green catalysts, such as sodium montmorillonite, sodium montmorillonite [pmim] Cl, and sodium montmorillonite [pmim] HSO_4_, were prepared to carry out the reaction. The three Na^+^MMT[pmim] HSO_4_ catalysts exhibited excellent activity with 100% conversion of the reactant with better yield (Scheme 39). The reaction between an aromatic aldehyde 151 and dimedone 152 with the 2-aminobenzimidazole 153 in the presence of Na^+^MMT [pmim] HSO_4_ Brønsted acidic ionic liquid supported MMT clay heterogeneous catalyst under the without solvent condition at 110 °C provided excellent product tetrahydro benzimidazo quinazoline 154. When the reaction was performed in different catalytic mediums using different solvents, they provided lower yields than the Na^+^MMT [pmim] HSO_4_.

**Scheme 40:**
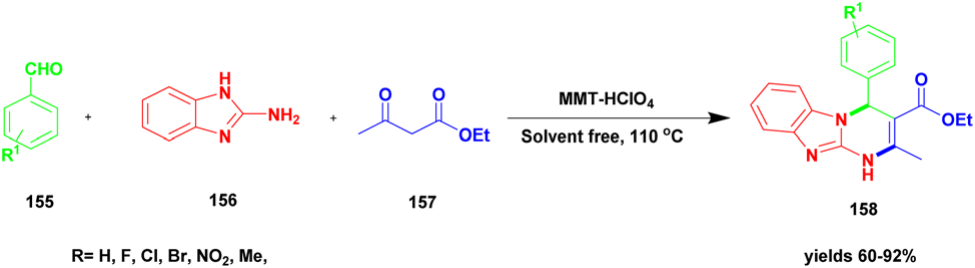
Synthesis of dihydropyrimidine derivatives using MMT-HClO_4_ catalyst.

Mashhadinezhad *et al.*^129^ documented a Na^+^ montmorillonite perchloric acid heterogeneous catalyst employed for the synthesis of dihydropyrimidine-attached heterocyclic compounds using a solvent-free green chemistry method. The dihydropyrimidine derivative compounds exhibit excellent biological applications used against various disorders in medicinal fields. The preparation techniques have some drawbacks, and to avoid these drawbacks, the reaction was conducted using a heterogeneous catalyst and a greener method. The authors performed the reaction under natural Na^+^ – montmorillonite clay incorporated with a perchloric acid catalyst. The reaction between aldehyde 155 and benzimidazole 156 with ethylacetoacetate 157 in the presence of MMT-HClO_4_ catalyst under a solvent-free method provided the expected dihydrobenzimidazolo pyrimidine 158 derivatives with a better yield of up to 91% (Scheme 40). The same reaction carried out on a substituted substrate with the same MMT-HClO_4_ catalyst also provided a good yield. When the same reaction was carried out using a different catalyst, a lower yield was observed. The main merits of the prepared catalyst were easy recovery of the catalyst from the reaction mixture and reusability for another set of reactions without the loss of its catalytic activity. Even four cycles provided a better yield, and only 2% of product formation was decreased.

**Scheme 41:**
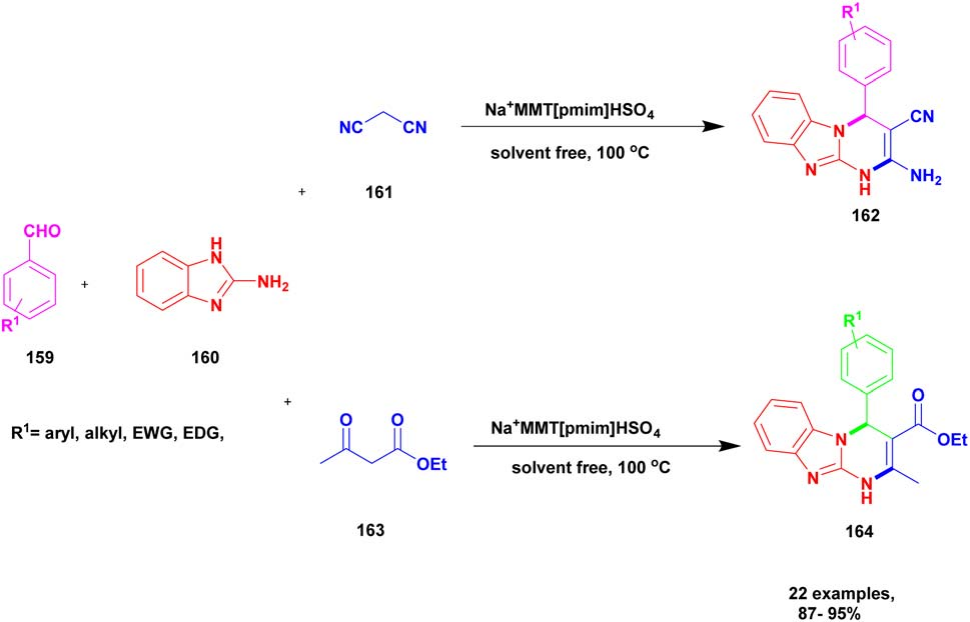
Construction of pyrimido-benzimidazole and pyrimido-benzimidazole-3-carboxylate using eco-friendly Na^+^MMT [pmim] HSO_4_ catalyst.

Makhsous *et al.*^130^ addressed a protocol for the construction of pyrimido [1, 2-*a*] benzimidazole moiety and pyrimido [1, 2-*a*] benzimidazole-3-carboxylate species using a solvent-free greener method with a brønsted acidic ionic liquid supported sodium montmorillonite clay heterogeneous catalyst. The pyrimido benzimidazole compounds have diverse biological activities used against various infections. Previously, executing this reaction under greener conditions had certain drawbacks. Hence, the authors introduced a Brønsted acid ionic liquid-supported sodium montmorillonite nanocomposite. The catalytic activity of the prepared catalyst was investigated by performing a reaction between an aromatic aldehyde 159 and 2-aminobenzimidazole 160 with cyanoacetonitrile 161 in the presence of Na^+^ MMT [pmim] HSO_4_ heterogeneous catalyst under solvent-free conditions at 100 °C. The expected pyrimido [1, 2-*a*] benzimidazole 162 was obtained with a better yield. The reaction was performed using ethylacetoacetate 163 instead of cyanoacetonitrile use, and the product tetrahydrobenzimidazo [1, 2-*a*] quinazoline-1-2(*H*)-one 164 derivative was obtained in a very good yield (Scheme 41). The same reaction was carried out in different solvents, and less product was formed. They require a longer time compared with solvent-free Na^+^MMT [pmim]HSO_4_ catalyst medium, and it provides a better yield of up to 95%.

#### 2.4.5 Silver nanoparticle-supported montmorillonite clay catalyzed carbon–nitrogen bond formation

**Scheme 42:**
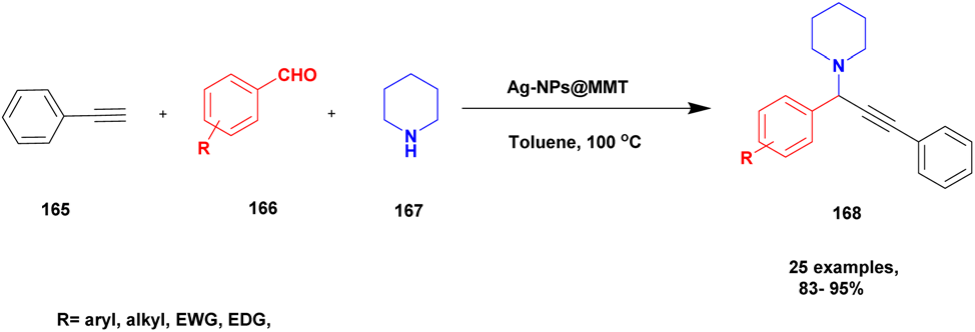
Synthesis of propargylamine derivative using Ag-NP-MMT catalyst.

Borah *et al.*^131^ reported a silver nano particle-stabilized montmorillonite heterogeneous catalyst for the synthesis of propargylamine derivatives. The propargylamine was an excellent intermediate for the synthesis of the biologically active drug compound. Initially, in the last decades, metal and metal nanoparticles have been used as catalysts for the synthesis of propargyl amine derivatives. It had some drawbacks, such as pre-long reaction time, expensive catalysts and solvents, and difficulty recovering the catalyst. To avoid these limitations, the reaction was carried out with a heterogeneous catalyst. Montmorillonite clay has excellent activity, is easily available, is eco-friendly, and does not affect nature and the environment. The authors introduced silver nano particle-stabilized montmorillonite clay, and the catalytic activity of the heterogeneous catalyst was investigated. The reaction between aromatic phenylene acetylene 165 and aldehyde 166 with secondary amine 167 in the presence of silver nano particle-stabilized montmorillonite heterogeneous catalyst and toluene solvent at 100 °C provided propargyl amine 168 products with a better yield of up to 95% (Scheme 42). When the reaction was carried out on different substituted substrates, no changes occurred in the product formation. The catalyst can be easily recycled, washed again and reused for another set of reactions. After the fourth cycle, the product formation decreased.

#### 2.4.6 Acid-activated montmorillonite catalyst using carbon–nitrogen bond formation

**Scheme 43:**
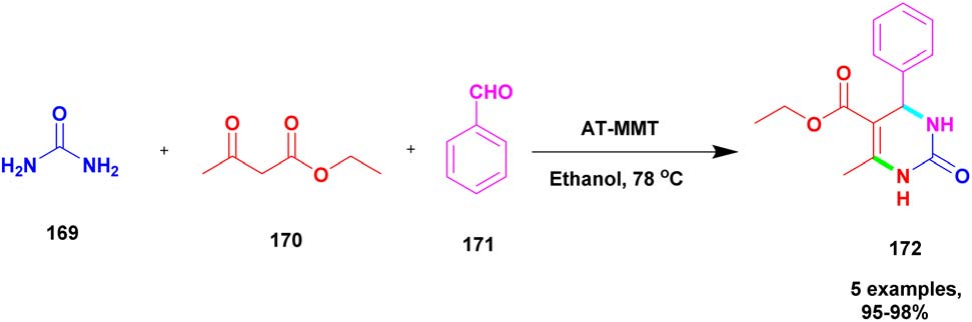
Synthesis of dihydropyrimidine-1(2*H*)-one using AT-MMT catalyst.

Phukan *et al.*^132^ reported that a heterogeneous mesoporous catalyst was used in the Biginelli reaction for the synthesis of a 3, 4-dihydropyrimidine-2(1*H*)-one. Dihydropyrimidine naturally occurs in some sea products. It exhibits excellent diversity applications in the medicinal field and is used against some diseases. Initially, the heterogeneous catalyst and some metal catalysts were used to synthesize a compound, but it had some drawbacks, such as a long reaction time, expensive catalyst and solvents, need for a specific environment, and the separation of catalyst from reaction mixture required a long time. To avoid these limitations, the reaction was conducted using an eco-friendly greener method. The authors carried out the reaction using naturally available montmorillonite clay, which was very cheap and did not affect nature and the environment. The acid-activated montmorillonite clay exhibits excellent activity owing to its large surface area with small mesoporous diameter; here hydrochloric acid was used to activate the MMT catalyst. The reaction between diamminoketone 169 and β-ketoester 170 with aromatic aldehyde 171 in the presence of acid-activated MMT clay heterogeneous catalyst in ethanol solvent medium provided very good 3,4-dihydropyrimidine-one 172 product with an excellent yield of up to 98% (Scheme 43). When the reaction was carried out in different substitute substrates, a better yield was obtained. Additionally, we can easily separate the catalyst from the reaction mixture and reuse it for another set of reaction up to four cycles with excellent yield; after the fourth cycle, the catalytic activity decreased.

#### 2.4.7 Zwitter ionic sulfamic acid-functionalized montmorillonite clay catalyzed C–N formation

**Scheme 44:**
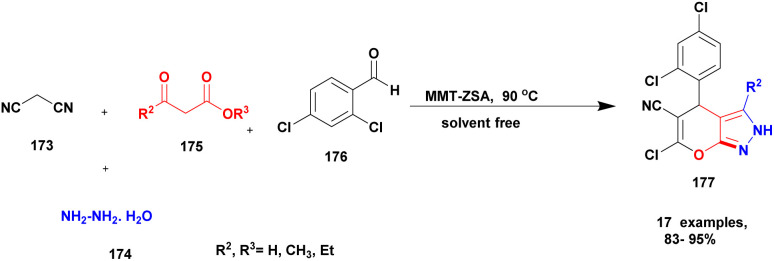
Preparation of pyrano pyrazoles using the MMT-ZSA catalyst.

Safari *et al.*^133^ reported a document for the preparation of pyrano pyrazoles using a multicomponent integrated greener method with a zwitterionic sulfamic acid-supported nano clay heterogeneous catalyst. The pyran-substituted compounds play a predominant role in the synthesis of medicinally and biologically active compounds that are used against various biological problems. Earlier method preparation of the pyran derivatives, the metal catalysts, organic catalysts and heterogeneous catalysts was used. It had some drawbacks, such as prolonged reaction time, expensive solvents and catalysts, very poor yield, and a very tedious process carried out to remove the catalyst from the reaction mixture. These limitations are avoided by performing the reaction using a greener method. Montmorillonite is a naturally available clay mineral. It acts as a better catalyst with both Lewis acid and Brønsted acid nature and a high surface area. The surface-modified MMT with superior catalytic activity provides better yield. The authors introduced a zwitterionic sulfamic acid-functionalized montmorillonite heterogeneous catalyst. It exhibits excellent activity. The reaction between malononitrile 173, hydrazine 174 and β-ketoester 175 with aromatic aldehyde 176 under solvent-free one-pot conditions provided dihydropyrano pyrazole 177 derivative with a better yield of up to 95% (Scheme 44). When the same reaction was carried out with a solvent and different catalysts, lower yield of product was obtained compared to that of the ZIA-MMT catalyst. The major advantage of the reaction was the ease of recovery of the catalyst from the reaction mixture using a simple filtration technique and reuse for another set of reactions without loss of its catalytic activity. After the fourth cycle, the yield of the product decreased by 3%, and no separation technique was required.

#### 2.4.8 Heteropolyacid-supported natural clay using carbon–nitrogen bond formation

**Scheme 45:**
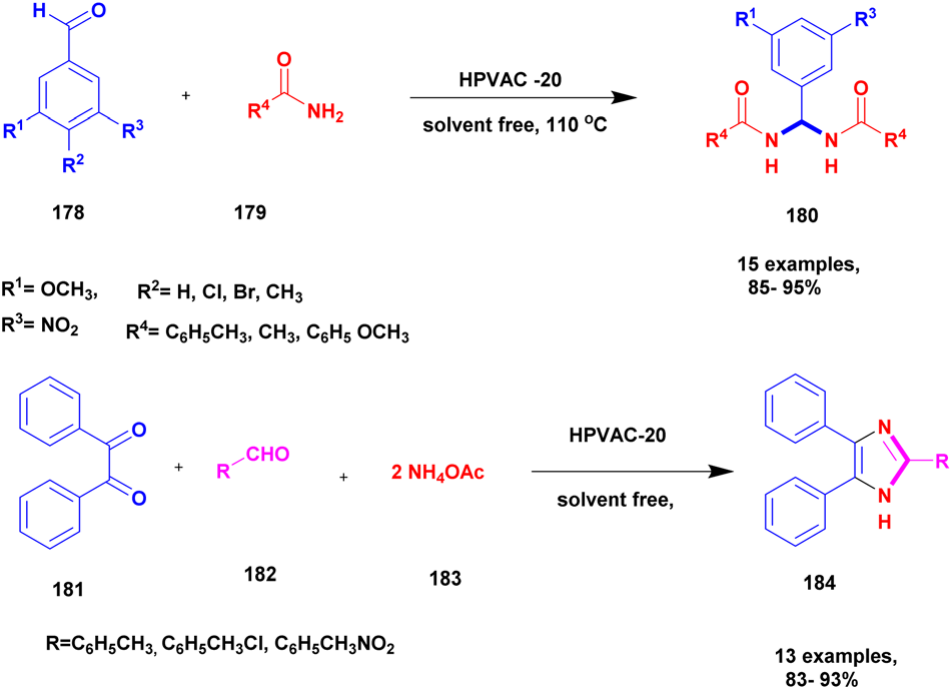
Construction of *N*, *N*-alkylidene bisamide moiety using HPVAC-20 catalyst.

Selvakumar *et al.*^134^ reported an extension of the above work to synthesize *N*, *N*-alkylidene bisamide and imidazole using the same HPVAC incorporated with a natural clay heterogeneous catalyst. Initially, when the reactions were carried out using different catalysts, only a poor yield was obtained and recovery of the catalyst was difficult. The author reported a novel natural clay-based catalyst to overcome these limitations. The reaction between aromatic aldehyde 178 and amide 179 condensation reaction yielded *N*, *N*- alkylidine bisamide 180. For another reaction between diphenylethanedione 181 and aromatic aldehyde 182 with ammonium acetate 183 in the presence of an HPVAC-20 heterogeneous catalyst, the condensation reaction yielded tri-substituted imidazole 184 compounds as the expected product (Scheme 45). The major advantages of the HPVAC-20 natural clay catalyst are its low cost, ease of separation from the reaction mixture, and reusability.

**Scheme 46:**
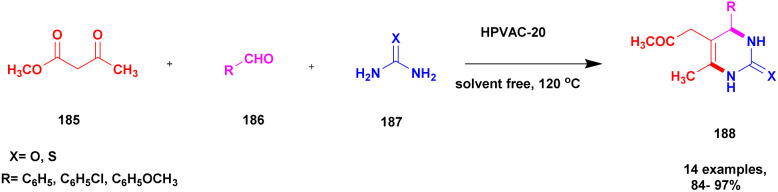
Synthesis of 3, 4 dihydropyrimidine/thione derivatives.

Selvakumar *et al.*^135^ reported a natural clay-catalyzed heteropolyacid-supported C–N bond formation reaction. One-pot synthesis of compound 3, 4-dihydro pyrimidone and thiones was carried out. Here, the HPVAC-20 catalyst was used with natural clay, and it exhibited excellent catalytic activity compared to the metal catalyst. Initially, the reaction was conducted with various combinations of heteropoly acid supported with natural clay catalysts, and they provided less amount of yield compared to HPVAC-20. The condensation reaction between methyl acetoacetate 185, aldehyde 186, and urea/thiourea 187 occurred under solvent-free condition *via* a one-pot synthesis method in the presence of HPVAC-20 catalyst and provided 3,4-dihydropyrimidine-one 188 product with excellent yield of 91% (Scheme 46). When the same reaction was executed in the presence of solvent, a lower yield was obtained. The HPVAC-20 is an excellent catalyst, and it easily separates from the reaction medium using simple separation techniques without any change in catalytic activity and is reused for another reaction.

#### 2.4.9 Redbrick clay catalyzed carbon–nitrogen bond formation

**Scheme 47:**
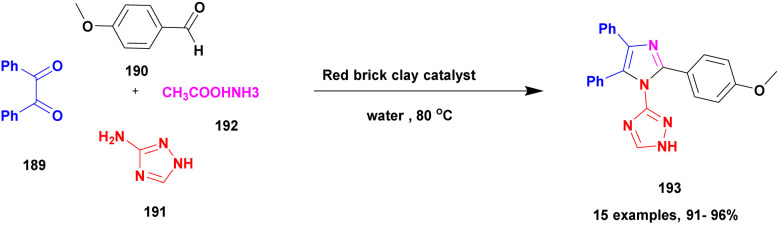
Preparation of tetrasubstituted imidazole using red brick clay catalyst.

Kerru *et al.*^136^ addressed a protocol for the synthesis of 1, 2, 4, and 5 – tetrasubstituted imidazole *via* red brick clay used as a heterogeneous catalyst. Nitrogen contains heterocyclic compounds that possess diverse applications in various fields, which include biological applications. The poly-substituted imidazole exhibited excellent activity. Clays are naturally occurring environmentally friendly materials with a non-hazardous nature. Nowadays, there are many clay materials used as catalysts. The authors carried out the reaction using red brick clay as a catalyst. The reaction between benzil 189 and aromatic aldehyde 190 with 1, 2, 4-triazole 3-amine 191 and ammonium acetate 192 in the presence of a red brick clay catalyst with water solvent using a greener method provided a very good coupled tetra substituted imidazole 193 product (Scheme 47). The same reaction was carried out with different solvent mediums and without solvent medium; the water solvent used method provided a very good yield of up to 96%. The advantages of the catalyst are its green and non-toxic nature, natural availability, easy recyclability and reusability for another set of reaction; even after five cycles the catalyst provides better yield with a negligible decrease in the product yield.

#### 2.4.10 KF-modified natural clay supported carbon–nitrogen bond formation

**Scheme 48:**
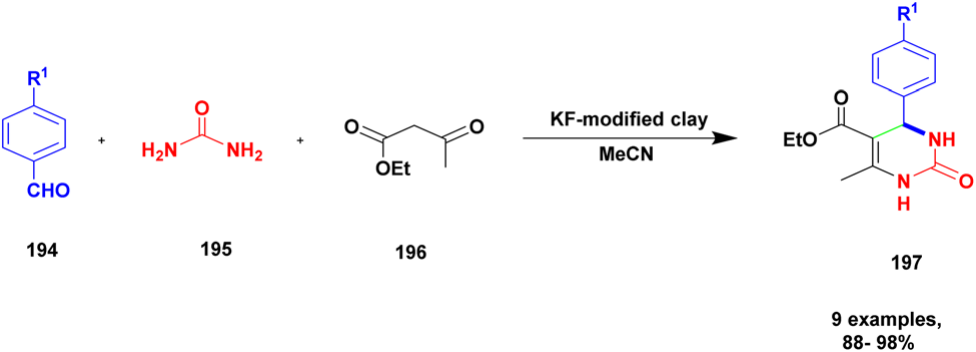
Synthesis of dihydropyrimidinone using natural KF-modified clay.

Bentahar *et al.*^137^ addressed a protocol for the synthesis of 3, 4-dihydropyrimidinone using a greener method with potassium fluoride-modified natural clay as a heterogeneous catalyst. The dihydropyrimidinone derivative compounds possess excellent biological properties and are used in the medicinal and pharmacological fields. The one-pot synthesis method is commonly used for the synthesis of dihydropyrimidinone. Initially, many homogeneous catalysts are used to prepare the compound, but they have some drawbacks. To avoid these demerits, a reaction was performed using a greener method. The authors introduced potassium fluoride as a natural clay catalyst, and it was easily available, a very cheap material, and a non-hazardous nature. The reaction between an aromatic aldehyde 194 and diaminoketone 195 with ethylacetoacetate 196 in the presence of KF-modified natural clay with methylcyanide solvent at 40 °C provided an excellent coupling product dihydropyrimidinone 197 with a better yield of up to 94% (Scheme 48). When the same reaction was carried out on different substituted substrates with electron-withdrawing or electron-donating groups, the parachloro-substituted aromatic aldehyde provided a very good yield of up to 97%. The mechanism of the reaction has been described as follows: initially, Knoevenagel condensation has taken place between aldehyde and acetate moiety. Then, simultaneously, aldol condensation occurred, and an activated alkene was formed. Next, the nucleophilic attack of the nitrogen and homogeneous catalysis occurred, followed by cyclization, and the expected product dihydropyrimidine was formed.

#### 2.4.11 Kaolin clay-supported carbon–nitrogen bond formation reaction

**Scheme 49:**
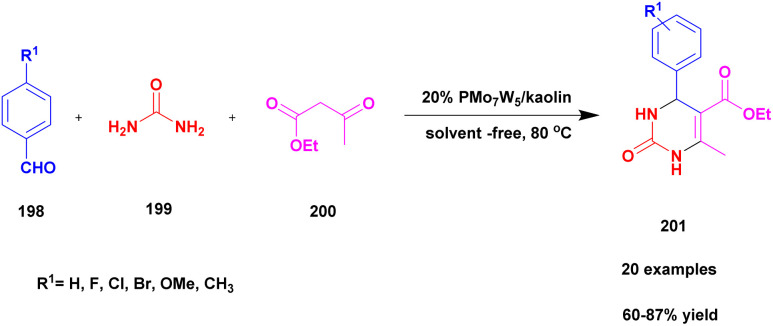
Synthesis of 3, 4-dihydropyrimidineone using PMo_7_W_5_/kaolin clay catalyst.

Aher *et al.*^138^ addressed a protocol for the preparation of 3, 4-dihydropyrimidinone *via* Biginelli reaction using a tungsten-substituted molybdophosphoric acid impregnated kaolin clay heterogeneous catalyst. The Keggin-type heteropoly acids possess an excellent Brønsted acidity and are used as catalysts in many reactions. The main drawbacks of heteropoly acids were poor surface area and fast reactivity. To avoid these problems, the supported material was introduced. Here, kaolin clay was easily available in nature and very cheap. It has a large surface area and excellent catalytic activity and is used as a heterogeneous catalyst in various chemical reactions. The acidified-kaolin clay possessed a wider surface area than pure kaolin clay, and the Keggin-type heteropoly acids were impregnated with solid kaolin clay to increase the catalytic activity and surface area. Initially, the same reaction was carried out using different catalysts. The kaolin-WMoPA provided a very good yield compared with them. The reaction between benzaldehyde 198 and urea 199 with ethyl acetoacetate 200 in the presence of PMoW/kaolin clay heterogeneous catalyst using the solvent-free method provided excellent product 3, 4-dihydropyrimidinone 201 with a very good yield of up to 95% (Scheme 49). When compared with other catalysts and solvent mediums, the kaolin-impregnated PMoW catalyst gives a better product in a short time at 80 °C temperature. Additionally, the catalyst can be separated from the reaction mixture and reused for another set of reactions without the loss of its catalytic activity.

#### 2.4.12 Red clay-supported carbon–nitrogen bond formation reaction

**Scheme 50:**
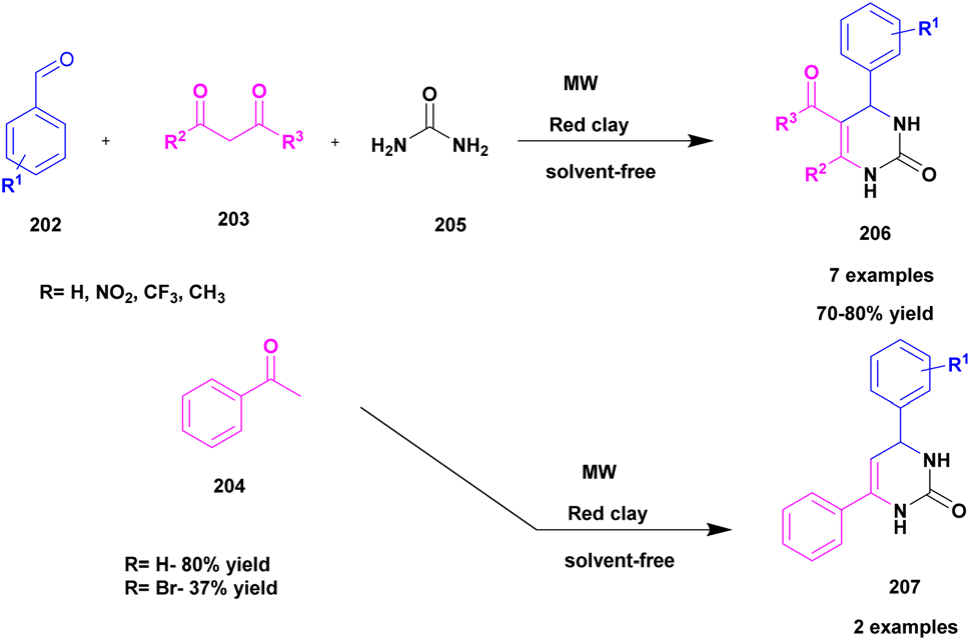
Synthesis of dihydropyrimidone derivatives 206 and 207.

Babar and his group^139^ examined the use of organic red clay as a mild catalyst for the microwave-assisted synthesis of Schiff's bases of dihydropyrimidones with aniline. Microwave irradiation provides a cleaner alternative to conventional organic synthesis processes, making it a more environmentally friendly option. The compounds were synthesized by reacting aniline at 400 watts. The synthesis of DHPM derivatives 206 and 207 was achieved by combining substituted aromatic aldehydes 202, ethyl acetoacetate 203 or acetyl acetone 204, urea/thiourea 205, and red clay as a catalyst using a simple multicomponent one-pot method (Scheme 50). The compounds were then combined with aniline to form their Schiff bases in a high yield (65–81%). The first phase in the formation of Schiff bases is the non-nucleophilic transformation of the amine, and the second step is a leftward shift in equilibrium. Several Schiff bases are synthesized most effectively at a slightly acidic pH. In this case, acidic red clay can act as a catalyst.

#### 2.4.13 White clay-supported carbon–nitrogen bond formation reaction

**Scheme 51:**
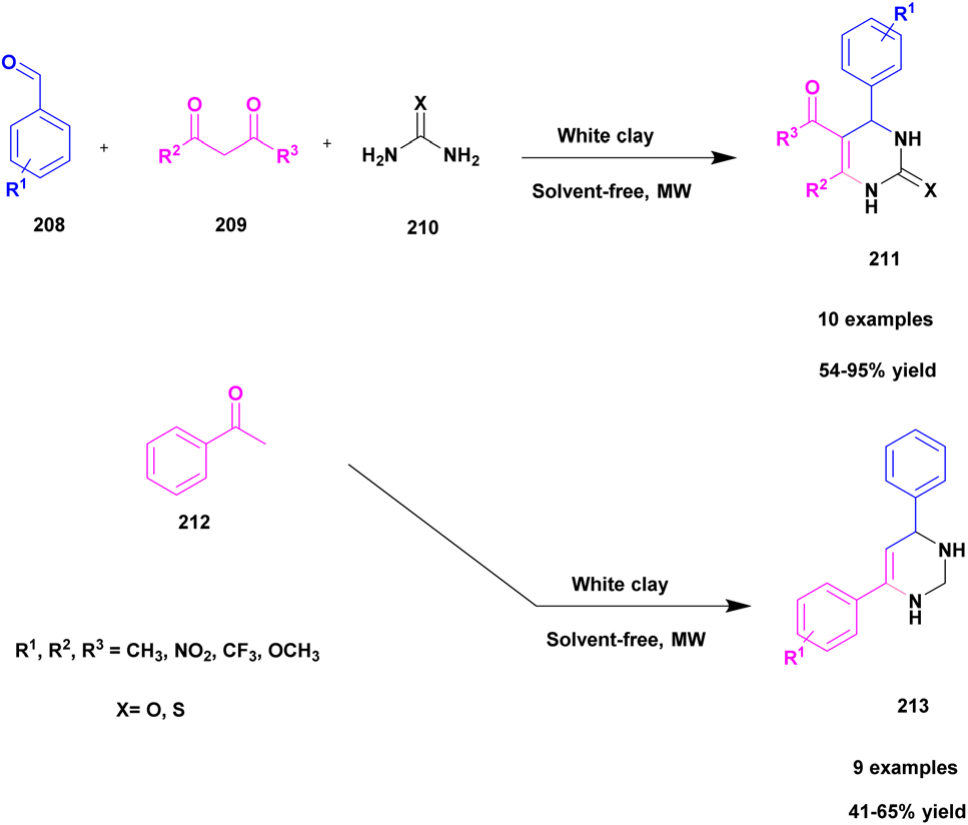
Synthesis of 3,4-dihydrpyrimidone derivatives using an organic white clay catalyst.

Babar and his research group^140^ reported a work on the synthesis of 3,4-dihydrpyrimidone derivatives using a microwave method with an organic white clay catalyst. White clay was collected from the west Indian region. The reaction carried out between aldehyde 208 and ethylacetoacetate 209 and urea 210 in the presence of white clay catalyst 0.2 g without a solvent by subjecting to microwave for 10 min at 250 W gave 3,4-dihydropyrimidine 211 product with 90% yield (Scheme 51). Instead of ethylacetoacetate and urea, the use of acetophenone 212 derivatives and thiourea gave 4,6-diphenyl-3,4-dihydropyrimidone 213 products with a 65% yield. Both reactions were carried out using different derivatives with electron donating and electron with-drawing substituents. The 3,4-dihydropyrimidine gave a good yield (36–95%), and the electron donating and unsubstituted compound (H, CH_3_, OC_2_H_5_) gave a good yield of 90–95%. The electron withdrawing group (3-NO_2_, Cl, F, Br, I, and CF_3_) attached substituents gave moderate 54–60% yield. Moreover, 3,4-disubstited OCH_3_ substituents gave a 36% yield, and a 4-OCH_3_ substituted reaction did not occur. The 4, 6-diphenyl-3, 4-dihydropyrimidone compound was also optimized with different derivatives, and the electron withdrawing substituents 4-Br and 4-NO_2_ gave a good yield of 61–65%. The electron donating 4-OCH_3_ and the unsubstituted group gave less yield of 40–43%. All these reactions were carried under microwave medium and gave product within 5–10 min using 0.2 g of white clay catalyst.

#### 2.4.14 General mechanism of the clay-supported carbon–nitrogen bond formation reaction

##### 2.4.14.1 Biginelli reaction

**Scheme 52:**
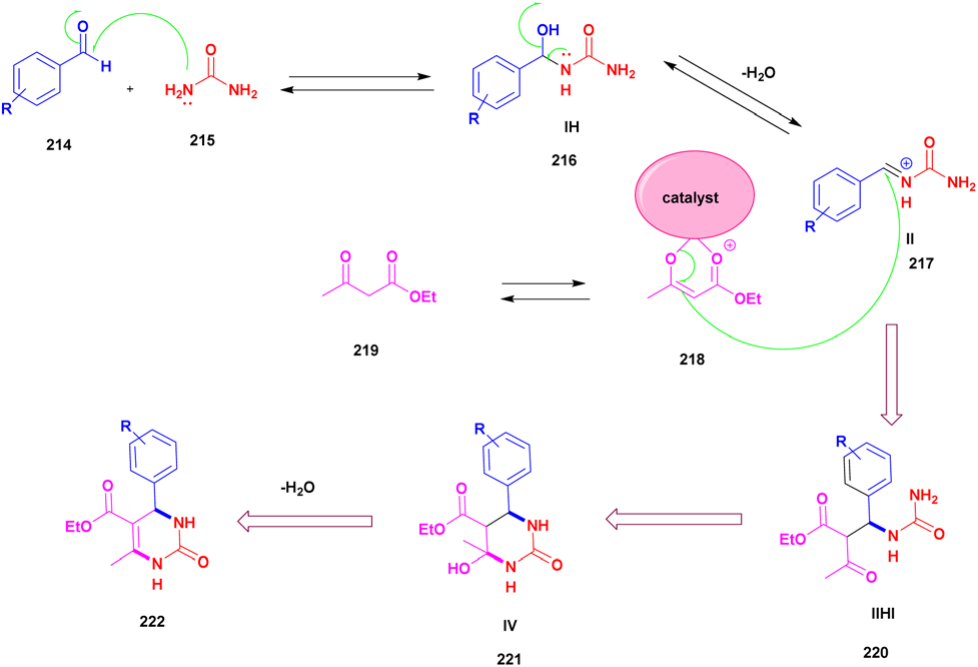
Plausible mechanism of Biginelli derivative formation.

In this review, most of the reactions discussed dihydropyrimidinone formation *via* the Biginelli reaction (Scheme 52). Initially, the heterogeneous clay catalyst activated the carbonyl group formation of the electrophilic carbon centre. The next step was the nucleophilic attack of urea on the electrophilic carbon centre activated with an iminium ion intermediate. Then, the iminium intermediate reacted with ethyl acetoacetate to form a stable intermediate III. The formed intermediate underwent intermolecular cyclization to form intermediate IV; finally, the expected Biginelli derivative product was formed.^141^

##### 2.4.14.2 A^3^ coupling reaction

**Scheme 53:**
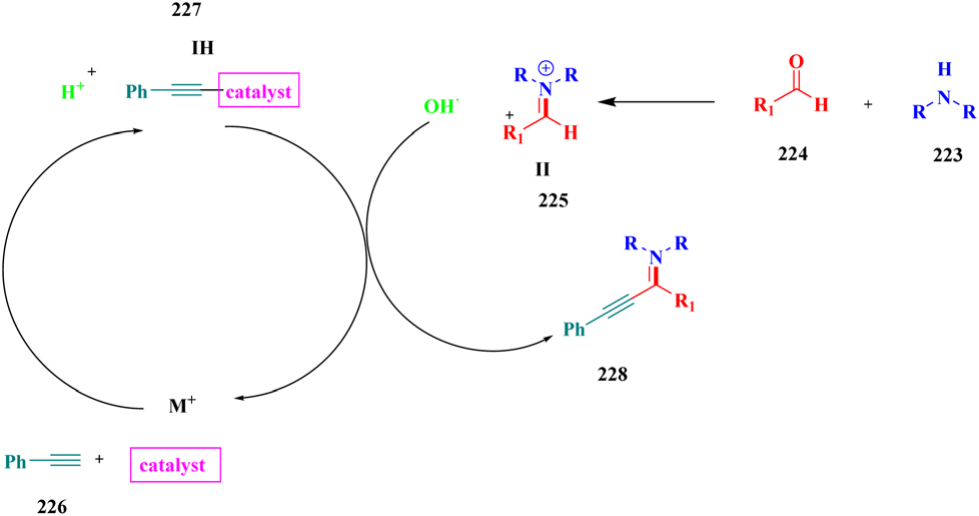
Plausible mechanism of A^3^ coupling reactions.

The A^3^ coupling reaction is one of the major C–N bond formation reactions, and the A^3^ coupled products have excellent activity in various fields (Scheme 53). The mechanistic pathway of the reaction with an initial condensation reaction takes place between the aldehyde and amine to form an iminium ion intermediate. Next, the formed intermediate reacted with acetylene in the presence of a catalyst, forming a three-component coupled C–N bonded product.^142^

##### 2.4.14.3 KA^2^ coupling

**Scheme 54:**
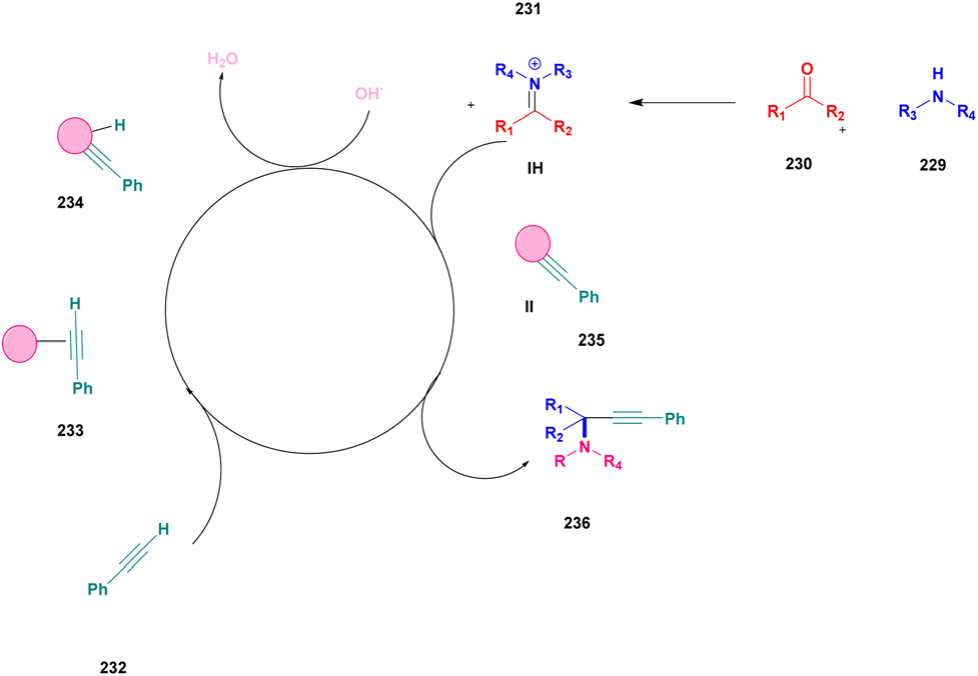
Plausible mechanism for KA^2^ coupling.

The KA^2^ coupling is the three-component coupling of aldehyde, amine and ketone that reacts to form a C–N bonded product (Scheme 54). The mechanism of KA^2^ coupling is similar to that of the A^3^ coupling; instead of aldehyde, the KA^2^ coupling ketone was used.^143^

## 3 Conclusions

In this review, we highlighted the activity of clay-based material for the synthesis of carbon–nitrogen bonded compounds from 2016 to date. Clays are easily available and low-cost catalytic materials; they exhibit excellent catalytic activity and are widely employed for diverse applications in organic synthesis. Over the past few years, clay-based catalysts have attracted synthetic organic chemists. The modified clay catalyst achieves a better yield for different types of organic reactions. The metal, metal oxides, inorganic complexes, and some organic compounds are used to modify clay materials because they have versatile activity to provide highly selective products. Many multicomponent reactions are performed using various clay catalysts, among which the Biginelli reaction and A^3^ coupling reaction are important for the synthesis of C–N bonded organic products. The metal nanoparticle incorporated and Keggin-type heteropoly acid supported clay catalysts are used for the construction of several carbon–nitrogen bonded heterocyclic compounds. Clay-based catalysts are widely used in organic synthesis owing to their excellent catalytic activity. This kind of catalyst is emerging as an effective one as evident from the literature aforementioned. Though there are some drawbacks to clay as a catalyst, its excellent catalytic activity overshades them, which is of interest to various researchers working in this field. The present review emphasizes various modified clays, such as bentonite, montmorillonite, hydrotalcite, halloysite, red brick, and natural clay supported or catalyzed carbon–nitrogen bond formation reactions to synthesize the selectively coupled product.

## Conflicts of interest

There are no conflicts to declare.

## Supplementary Material
